# Atomistic molecular dynamics simulations of bioactive engrailed 1 interference peptides (EN1-iPeps)

**DOI:** 10.18632/oncotarget.25025

**Published:** 2018-04-27

**Authors:** Neha S. Gandhi, Pilar Blancafort, Ricardo L. Mancera

**Affiliations:** ^1^ School of Mathematical Sciences and Institute for Health and Biomedical Innovation, Queensland University of Technology, Gardens Point Campus, Brisbane QLD 4000, Australia; ^2^ Cancer Epigenetics Group, The Harry Perkins Institute of Medical Research, Perth WA 6009, Australia; ^3^ School of Pharmacy and Biomedical Sciences, Curtin Health Innovation Research Institute and Curtin Institute for Computation, Curtin University, Perth WA 6845, Australia

**Keywords:** Engrailed homeodomain, transcription factor, iPep, breast cancer, molecular dynamics

## Abstract

The neural-specific transcription factor Engrailed 1 - is overexpressed in basal-like breast tumours. Synthetic interference peptides - comprising a cell-penetrating peptide/nuclear localisation sequence and the Engrailed 1-specific sequence from the N-terminus have been engineered to produce a strong apoptotic response in tumour cells overexpressing EN1, with no toxicity to normal or non Engrailed 1-expressing cells. Here scaled molecular dynamics simulations were used to study the conformational dynamics of these interference peptides in aqueous solution to characterise their structure and dynamics. Transitions from disordered to α-helical conformation, stabilised by hydrogen bonds and proline-aromatic interactions, were observed throughout the simulations. The backbone of the wild-type peptide folds to a similar conformation as that found in ternary complexes of anterior Hox proteins with conserved hexapeptide motifs important for recognition of pre-B-cell leukemia Homeobox 1, indicating that the motif may possess an intrinsic preference for helical structure. The predicted NMR chemical shifts of these peptides are consistent with the Hox hexapeptides in solution and Engrailed 2 NMR data. These findings highlight the importance of aromatic residues in determining the structure of Engrailed 1 interference peptides, shedding light on the rational design strategy of molecules that could be adopted to inhibit other transcription factors overexpressed in other cancer types, potentially including other transcription factor families that require highly conserved and cooperative protein–protein partnerships for biological activity.

## INTRODUCTION

Studies of breast carcinomas have established the role of transcription factors (notably those that contain developmental homoedomains) to be highly expressed in cancers and as drivers of cancer initiation, disease recurrence and resistance to treatment [1]. Expression of the homeobox-containing genes Engrailed 1 (EN1) and 2 (EN2) has been found - during development of the central nervous system in humans and is thought to be crucially involved in the anatomic organisation of the mid-gestational medulla and cerebellum [2]. The functional significance of the overexpression of Engrailed members in cancers, particularly basal-like breast cancers, has recently been highlighted [3]. Cancer patients with high EN1 expression experience the lowest relapse-free survival rate, indicating the existence of an association between high EN1 expression and resistance to conventional cancer treatment such as chemotherapy. Transcription factors, unlike other molecular cancer targets, have largely remained “undruggable” due to a lack of small molecular binding pockets. Recently, interference peptides (EN1-iPeps) that selectively inhibit EN1 activity have offered a novel route for the treatment of aggressive basal-like triple negative breast carcinomas. The nanoparticle-encapsulated iPep has also been selectively targeted to basal-like cancerous cells in combination with anti-cancer drug docetaxel and doxorubicin to produce a highly synergistic pharmacological activity [4].

Experimental data indicates that EN1-iPeps prevent transcription through a dominant negative-like mechanism, inhibiting interactions between EN1 and its binding partners such as glutamyl-prolyl tRNA synthetase (EPRS) [3], and disrupting protein-protein interactions between transcription factor homeodomains necessary for cooperative DNA binding, as in the case of homologous EN2-PBX1 or HOXA9-PBX1 complexes [5]. The EN1-iPeps are derived from the EN1 transcription factor wherein the hexapeptide motif WPAWVY is highly conserved and is shared by the homeodomain superfamily of transcription factors. The N-terminus of these peptides is engineered with the cell penetration/nuclear localisation sequence (CPP/NLS) KKKRV, which is present in the Simian Virus 40 (SV40) large T-antigen and is necessary for the internalisation of the peptide through plasma and nuclear membranes [6]. The full-length EN1-iPep (Peptide 1) consists of 29 residues (including CPP) and produces strongly reduced viability in a dose-dependent manner with an IC_50_ of 17.5 μM in a SUM149PT cell-based assay. A shorter EN1-iPep (Peptide 2) lacking the less evolutionarily conserved five N-terminal residues and two C-terminal residues of the full-length EN1-iPep is more potent than Peptide 1, with a lower IC_50_ of 9.28 μM. Upon mutation of two tryptophan residues of the hexapeptide motif to alanine, the resulting EN1-iPepmut (Peptide 3) binds poorly to EPRS (IC_50_ > 50 μM), suggesting that the tryptophan residues are required for inhibiting cancer cell growth. A similar approach has been taken previously for the design of the synthetic peptides HXR9 and CXR9, which act as a competitive antagonists of the interaction between Hox proteins and their PBX co-factor [7].

Structural studies have been carried to understand the role of the conserved pentapeptide region (F/Y-P-W-M-R/K) within the hexapeptide region of Hox proteins in the interaction and modulation of DNA binding activity by Pbx1 and related proteins [8–10]. Crystal structures of the ternary complexes of Hox-DNA-Pbx1 suggest that the hexapeptide motif forms a 3_10_-helix that packs against the PBC (a member of the three-amino-acid loop extension superclass of homeodomain proteins) homeodomain and inserts the conserved tryptophan into the hexapeptide binding pocket [11, 12]. Peptides taken from a truncated full-length protein do not normally exhibit stable conformations in water solution. NMR and molecular dynamics (MD) simulations of peptides containing this hexapeptide motif show, however, that these small truncated peptides are capable of folding into stable turn structures that are equivalent to the structure of this region in the ternary complex of HOX-DNA-PBX [13–15].

To date several biophysical experimental and molecular modelling studies have been carried out to characterise the structure of engrailed homeodomains [16–20], except for the N-terminal extension (or EH-2 domain [21]), which consists of the hexapeptide motif. The structural characterisation of the EH-2 domain in EN1 and EN2 by experimental techniques has been limited by their intrinsic disorder in solution. Therefore, in this study we report scaled MD (sMD) [22] simulations of various EN1-iPeps to characterise their structure and dynamics in aqueous solution and define the structural basis for their specificity and biological activity.

## RESULTS AND DISCUSSION

### Secondary structure analysis

**Figure 1:**
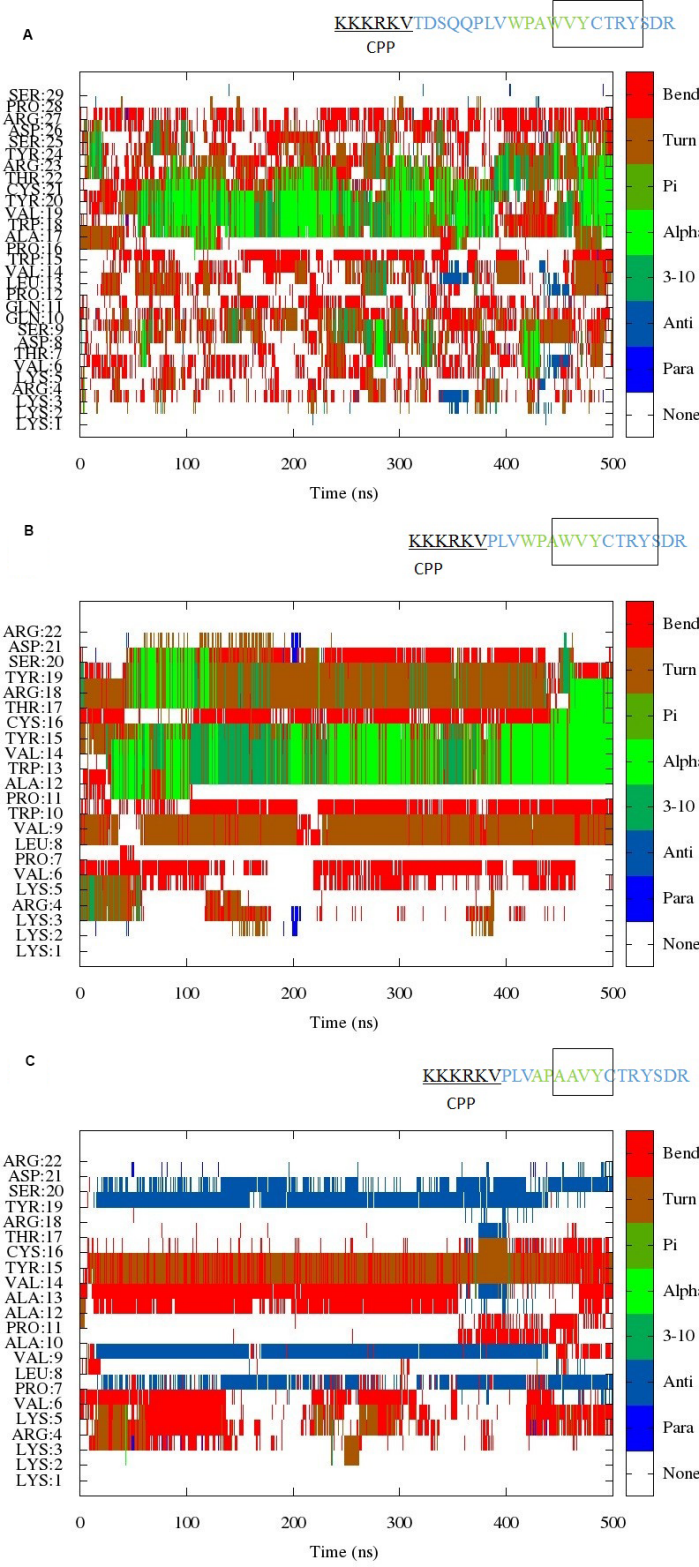
(**A**) Evolution of the secondary structure from scaled MD simulation of EN1-iPep (Peptide 1). The results indicate the presence of helical/turn conformation near the C-terminus of the peptide. A rectangular box in the sequence indicates the presence of stable secondary structure. (**B**) Evolution of the secondary structure from scaled MD simulation of Peptide 2. The results indicate the presence of helical/turn conformation near the C-terminus of the peptide. A rectangular box in the sequence indicates the presence of stable secondary structure. (**C**) Evolution of the secondary structure from scaled MD simulation of Peptide 3. The results indicate the presence of turn/bend conformation within the hexapeptide motif upon mutation of tryptophans to alanine. The presence of a region in the sequence with turn/bend conformation is indicated by a rectangular box.

Simulations of all three peptides were initiated from random coil structures. Figure 1 shows the evolution of secondary structure in the simulation of Peptides 1–3 as a function of time. The hexapeptide motif in the simulations of Peptides 1 and 2 shows a tendency to adopt turn/helical conformations (Figure 1A and 1B). In addition, a turn conformation is observed preceding the hexapeptide motif, which is very stable in the simulation of the shorter EN1-iPep 2 (i.e. Peptide 2). Peptide 2 is generated by truncation of five residues in Peptide 1 and analysis of the secondary structure (DSSP) of this shorter peptide clearly demonstrates a much more stable and well-defined conformation. This is further explained by the formation of hydrogen bonds, as discussed further below. By contrast, simulation of Peptide 3 revealed a tendency to fold into beta strands, such that the hexapeptide motif forms a bend-turn resulting in a β-hairpin structure (Figure 1C). Interestingly the cell penetrating sequence is predicted to be disordered for most of the time in all peptides.

### Hydrophobic interactions and hydrogen bond analysis

Members of the HOX family, e.g. HOXB1, have two aromatic residues in the hexamotif sequence TFDWM. Interactions between the aromatic residues phenylalanine and tryptophan were found to stabilise the folded state of the hexapeptides of HoxB1 in water [14, 15]. In the crystal structure (PDB code 1B72), the distance between the Cβ of the aromatic residues was found to be 5.87 Å [12]. The EN1-iPep hexamotif consists of three aromatic residues, i.e. two tryptophans and a tyrosine. Furthermore, there is an additional tyrosine after the hexapeptide sequence. The distances between Cβ of these aromatic residues were monitored in all of the simulations (see Table 1) and the cut-off distance was selected based on previous MD study [15].

**Table 1:** Interactions between aromatic residues observed in sMD simulations with ʎ = 0.7

Residue pairs	Peptide 1 (%)^*^	Peptide 2 (%)^*^	Peptide 3 (%)^*^
Trp15-Trp18	15.7	14.8	-
Trp18-Tyr20	8.6	4	-
Tyr20-Tyr24	20	10	negligible

^*^indicates data from biased simulations. The numbering is based on the sequence of peptide 1. Simulation time percentages correspond to distances less than 6.5 Å between Cβ-carbons on side chains in the respective aromatic residues.

In the case of the HOXB1 structure, the aromatic Phe and Trp are separated by a negatively charged Asp in the hexamotif. In the case of the EN1-iPeps, the two Trp residues are spaced two residues apart and, as a consequence, lack π-π interactions between them. Monitoring of the chi1 torsion of Trp18 and during half of the time in the simulations of Peptide 1 and Peptide 2 the average angle was 61°, in agreement with the value of the chi1 torsion found in the HOXB1-PBX-DNA complex [12]. The average value of 61° of the chi1 torsion angle appears to correlate with the presence of helical conformation in the hexapeptide region and the occurrence of CH- π interactions (as discussed below) (see Supplementary Figures 1 and 2). The mutant Peptide 3 only has two aromatic residues and the average distance between Tyr20 and Tyr24, and between the alanines in the hexapeptide motif, were found to be >13 Å and >7 Å, respectively.

Aromatic-proline interactions can occur locally in the tertiary structures of proteins and also - in intermolecular protein-protein interactions. Proline and aromatic residues can interact favourably with each other due to the hydrophobic effect and the interaction between the π aromatic face and the polarised C-H bonds (i.e. a CH-π interaction). CH-π interactions were found to stabilise secondary structure in series of Xaa-P peptides where Xaa is an aromatic residue [23]. Furthermore, the P-X-W motif is known to adopt a turn structure in the intrinsically disordered domain 2 of the NS5A protein [24], with trans being the dominant conformation. EN1-iPeps have both X-P and P-X-W motifs i.e. W-P and P-A-W in the hexapeptide motif. This suggests that it is likely that a turn-like conformation may exist in the hexapeptide motif due to aromatic and hydrophobic interactions. Molecular mechanics force-fields do not take into account electronic effects and hence cannot describe explicitly π -interactions (such as π -hydrogen bonds, π -stacking, π-OH and cation-π interactions); therefore, the presence of this interaction was determined by measuring distances between tryptophan and proline during the simulations. In the simulations of Peptides 1 and 2 (see Supplementary Figures 1 and 2, respectively), the average distance between the centroids of the rings in Pro16 and Trp18 were measured to be ~5 Å for nearly 50% of the simulation time, suggesting that these residues stabilise the formation of a helical conformation. For example, during the 150–200 ns range, the average value of the chi1 torsion angle of tyrosine 18/13 in Peptides 1 and 2 remained around 60° while, the distance between Pro16/11 and Tyr18/13 remained in the range 5–6 Å, and the dihedral angles within the hexapeptide correspond to a helical conformation in a Ramachandran plot. By contrast, during the 400–450 ns range in the simulation of Peptide 1, this trend was not observed.

A full analysis of all hydrogen bonding interactions was carried out for all simulations with biased potentials (Table 2). The hexapeptide motifs in Peptides 1 and 2 appear to have a strong tendency to form backbone hydrogen bonds between the carbonyl oxygen of residue i and the amide hydrogen of residue i+4 and i+3, with these hydrogen bonds supporting the α-helix and 3_10_ helix conformations. Such hydrogen bonding pattern is absent in Peptide 3 due to changes in interactions as a result of its corresponding mutations. There is a strong presence of ionic interactions between the side-chains of Asp26 and Arg27 (32%), and backbone hydrogen-bonding interactions between Tyr20/Cys21 and Tyr24 (12%) in Peptide 1, indicating that the turn/helix conformation extends to the C-terminus of the peptide, making it slightly longer than the one observed in the X-ray and solution structures of other known hexapeptides [11–13]. It is worth noting that truncation of residues in Peptide 1 gives rise to several hydrogen bonds in the N-terminus of Peptide 2, making it more stable than Peptide 1. In particular, Peptide 2 shows the presence of a backbone hydrogen bond between proline and tryptophan in the “PLVW” motif preceding the hexapeptide motif. Such hydrogen bonds are not observed in Peptides 1 and 3. There were no significant hydrogen bonds made by the C-terminal residues “PS”, therefore suggesting that truncation of the original EN1-iPep can be done without affecting key interactions, as it is indeed the case of Peptides 2 and 3. Various hydrogen bonds with Ala13 and Cys16 and/or between the residues from N- and C-termini of Peptide 3 indicate the formation of a well-defined secondary structure, such as a β-sheet connected with a β-turn. The N-terminus is in contact with the C-terminus due to the presence of a hydrogen bond between Asp21 with Arg4/Lys5 and with Arg22 and Arg4. The motif “X-P-X-X” where X is an alanine does not form any backbone or side chain interactions.

**Table 2:** Summary of hydrogen bonds in sMD simulations with ʎ = 0.7

Peptide 1		Peptide 2		Peptide 3	
Residue	(%)^*^		(%)^*^		(%)^*^
		Lys3:O - Lys5:H	5		
		Arg4:O - Val6:H	8	Arg4:O - Val6:H	16
				Lys5:O - Ser20:HG	10
		Val6:O - Arg4:HE	13		
		Val6:O - Arg4:HH11	6	Val6:O - Asp21:H	27
Thr7:O - Gln10:H	7				
				Leu8:O - Arg18:HH12	13
Gln11:OE1 - Gln11:H	5				
		Pro7:O - Trp10:H	12		
		Pro7:O -Arg4:HH22	9		
Pro12:O - Val14:H	9	Pro7:O - Val9:H	5		
Leu13:O - Trp15:H	7				
Pro16:O - Val19:H	15	Pro11:O - Val14:H	23	Pro11:O - Ala13:H	6
Pro16:O - Tyr20:H	12	Pro11:O - Tyr15:H	32		
Ala17:O - Cys21:H	16	Ala12:O - Cys16:H	17		
Ala17:O - Tyr20:H	6	Ala12:O - Tyr15:H	6		
Trp18:O - Thr22:HG1	20	Trp13:O - Thr7:HG1	6	Ala13:O - Cys16:H	6
Trp18:O - Thr22:H	11	Trp13:O - Ser20:HG	9		
Trp18:O - Cys21:H	6	Trp13:O - Cys16:H	8		
Val19:O - Arg23:H	10	Val14:O - Ser20:HG	29	Val14:O - Cys16:H	16
Val19:O - Thr22:HG1	8				
Val19:O - Thr22:H	6				
Tyr20:O - Tyr24:H	6	Tyr15:O - Arg22:HH12	12	Tyr19:O - Leu8:H	61
Tyr20:O - Arg23:H	6	Tyr15:O - Arg4:HH12	9	Tyr19:O - Val9:H	49
		Tyr15:O - Arg22:HH22	8		
Cys21:O - Tyr24:H	6	Cys16:O - Tyr19:H	41		
		Cys16:O - Ser20:H	34		
Asp26:OD1 - Arg27:HH21	8	Asp21:O - Arg4:HE	6	Asp21:O - Arg4:H	16
Asp26:OD1 - Arg27:HE	8	Asp21:OD1 - Arg4:HH21	5	Asp21:O - Lys5:H	6
Asp26:OD2 - Arg27:HH21	8	Asp21:OD2 - Arg22:H	5	Asp21:OD1 - Arg22:H	13
Asp26:OD2 - Arg27:HE	8	Asp21:OD1 - ARG22:H	6	Asp21:OD2 - Arg22:H	10
				Arg22:O - Arg4:HE	8
				Arg22:O - Arg4:HH22	8
				Arg22:O - Arg4:HH21	7
				Arg22:O - Arg4:HH12	6
				Arg22:OXT - Arg4:HH22	6
				Arg22:OXT - Arg4:HH21	6
				Arg22:OXT - Arg4:HE	6
				Arg22:OXT - Arg4:HH12	5

^*^Average hydrogen bonds obtained from biased simulations shown in percentage of simulation time (definition used for a hydrogen bond, distance and angle: <3.5 Å, <35°). Only hydrogen bonds with >5% occupancy are reported. O and H are the backbone atoms of residues whereas other atoms are from side chains. The table is arranged based on the acceptor atoms starting from the N-terminus.

### Chemical shift calculation

**Figure 2:**
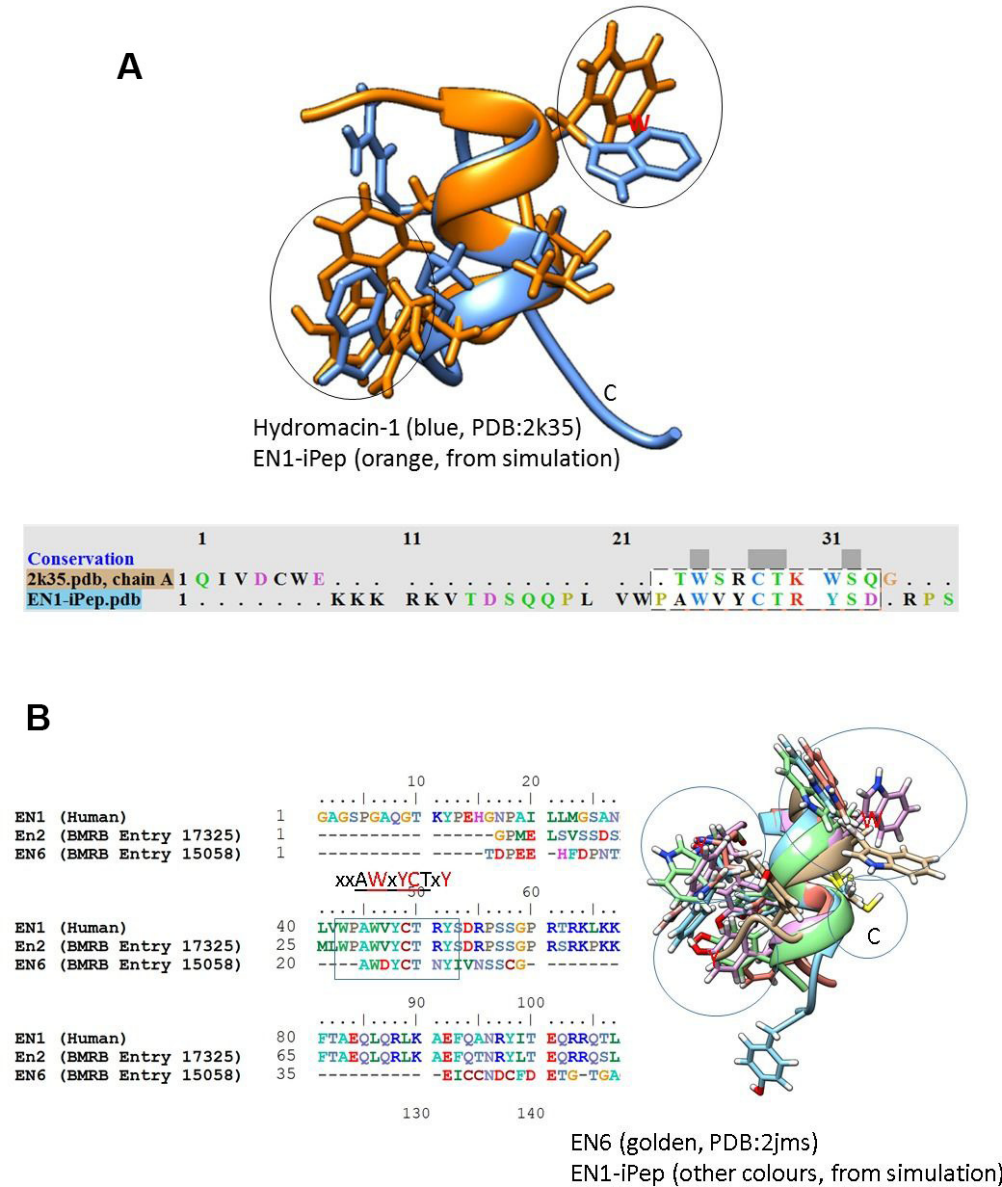
Structural alignment of conformations from simulations of Peptide 1 with (**A**) hydromacin and EN6 (**B**) NMR structures. The alignment indicates that the backbone conformations are similar in these structures, although the chi torsional angles (orientations) of aromatic residues are different. The sequence alignment of EN1 with EN2 and EN6 from the BMRB database indicates the presence of semi-conserved hexapeptide motifs (highlighted with a rectangular box).

SHIFTX2 was used to predict chemical shifts from each trajectory snapshot extracted from the simulations. Simulation averages were obtained for the 13Cα, 13C′, 1H′ and 15N chemical shifts (Table 3). In addition, a FASTA search was performed using the motif “PLVWPAWVYCTRYSDR” against the Biological Magnetic Resonance Data Bank (BMRB) to look for experimentally-derived NMR structural ensembles of HOX proteins, in particular with the corresponding hexapeptide motif. NMR assignments were found for the sequences of EN2 [25], hydramacin-1 [26] and pheromone En-6 [27] (BMRB entries 17325, 15739 and 15058, respectively) from chicken and Antarctic ciliate *Euplotes nobilii*. BMRB entry 17325 consists only of NMR assignments but no structure has been deposited, whereas structure assignments were carried out for BMRB entries 15739 (PDB code 23k5) and 15058 (PDB code 2jms). Sequence alignment of Peptide 1 with BMRB entry 15739 reveals that Tyr20 has been substituted by the basic residue arginine in hydramacin-1, whereas the structural alignment indicates that, despite the presence of a similar helical backbone in these structures, only one residue is conserved in the hexapeptide motif (Figure 2). The similarity in flanking sequence suggests that the sequence “C-T-R/K-Y/W-S” has a propensity to adopt a helical conformation.

**Table 3:** Predicted chemical shifts for common residues in the EN1-iPeps and relevant experimental values for EN2 and EN6

Residue	Peptide 1	Peptide 2	Peptide 3	EN2 (BMRB 17325)	EN6 (BMRB 17325)^++^
Trp15 C	174.187	174.367	^**^176.358	174.108	
Pro16 C	177.066	177.259	176.274	176.932	
Ala17 C	178.423	178.906	175.131	178.28	
Trp18 C	177.166	177.457	^**^175.704	176.719	
Val19 C	176.793	176.694	176.658	175.99	
Tyr20 C	176.539	176.029	177.269	175.92	
Cys21 C	175.086	175.486	177.088	174.671	
Thr22 C	174.734	175.478	176.238	174.527	
Arg23 C	175.927	176.318	175.166	176.054	
Tyr24 C	175.519	175.588	174.365	176.034	
Trp15 CA	55.4384	55.6216	^**^50.8622	54.622	
Pro16 CA	62.9905	63.1574	62.7129	62.723	
Ala17 CA	53.8897	54.5069	53.0646	53.799	
Trp18 CA	58.9418	59.2097	^**^51.8998	57.687	
Val19 CA	64.2555	64.1984	63.1707	62.947	
Tyr20 CA	59.5015	59.273	59.1239	58.107	
Cys21 CA	59.9428	59.0127	58.3381	58.637	
Thr22 CA	63.0689	64.3088	62.3337	62.284	
Arg23 CA	56.6999	57.8076	56.4246	56.234	
Tyr24 CA	58.1924	58.3568	57.3433	57.859	
Trp15 N	121.793	121.3007	^**^128.4233	121.901	
Ala17 N	123.947	123.8633	122.8543	122.547	
Trp18 N	118.217	117.5775	^**^120.9897	116.231	
Val19 N	118.227	117.3225	120.0067	120.895	
Tyr20 N	118.952	118.5206	121.0234	119.387	
Cys21 N	117.118	117.7995	116.9032	115.962	
Thr22 N	113.212	116.6971	116.6564	122.539	
Arg23 N	120.524	119.7902	123.7378	120.769	
Tyr24 N	118.855	117.0735	121.2028	121.901	
Trp15 H	7.75571	7.6096	^**^8.1919	8.227	
Ala17 H	8.26636	8.3523	8.2259	7.606	8.751
Trp18 H	8.12016	8.139	^**^7.7496	7.727	9.185
Tyr20 H	7.6936	7.1247	8.1622	7.955	8.669
Cys21 H	8.01274	7.7587	8.1626	8.085	9.155
Thr22 H	7.86433	7.8624	7.8086	8.086	7.986
Arg23 H	7.88829	8.3561	8.3271	8.126	8.751
Tyr24 H	7.83344	8.1223	8.3046	8.227	8.751
Trp15 HE1	10.0693	10.02	^**^N/A	10.017	N/A
Trp18 HE1	10.0919	10.0093	^**^N/A	10.186	9.860

^*^Numbering is based on the sequence of Peptide 1. ^**^Aromatic residues are substituted by alanine. ^++^Only 1H chemical shifts are available in the NMR-STAR format for EN6. Chemical shifts were calculated from biased simulations.

**Figure 3:**
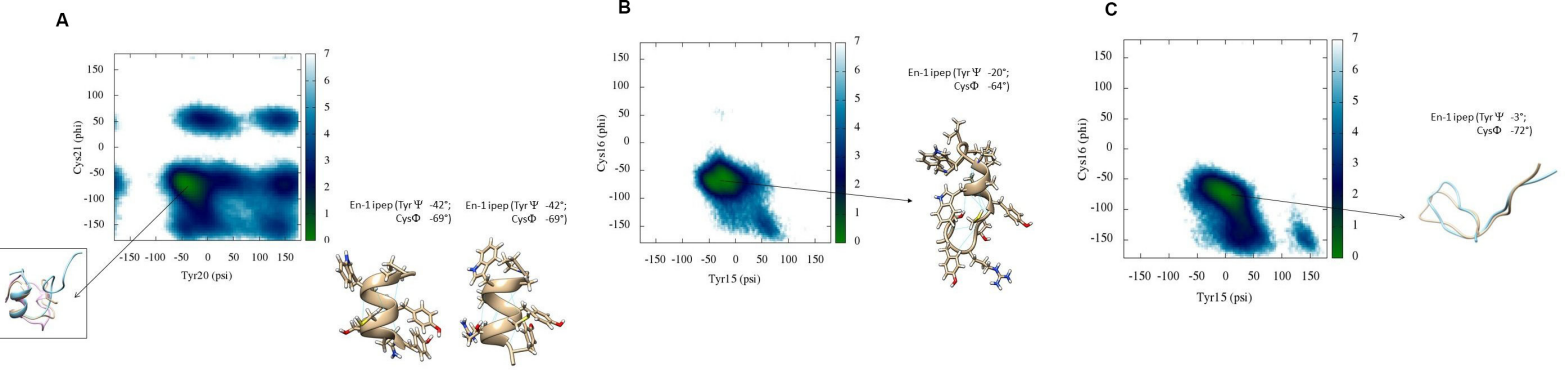
(**A**) Backbone torsional free energy landscape for Peptide 1. Peptide 1 shows a well-defined conformation in its C-terminus whereas the N-terminus is mostly disordered. The free energy minimum (kcal/mol) is located in a region where Tyr20 and Cys21 have psi and phi torsions between 0° to –70° and –50° to –100°, respectively. (**B**) Backbone torsional free energy landscape for Peptide 2. A representative conformation of the peptide near the free energy minimum is shown. (**C**) Backbone torsional energy landscape for Peptide 3. Residues Tyr20 and Cys21 only populate two regions in the Ramachandran plot, compared to Peptide 1. The lowest free energy minimum (kcal/mol) is located between 50° to –50° for psi and –50° to –100° for phi, respectively. These results are from scaled MD simulations with ʎ = 0.7.

The sequence alignment derived using ClustalW of full-length EN1 from human, EN2 from chicken and EN6 from *Euplotes nobilii* is shown in Figure 3. EN6 is divergent from EN2 and EN1, while the hexapeptide motif is semi-conserved. Based on this alignment, chemical shift assignments are only reported for residues 15–24 from the EN1-iPeps and compared with the experimentally-obtained chemical shifts of EN2 and EN6.

The amide chemical shifts of -all residues are higher in EN6 compared to the other peptides. This may be attributed to the fact that cysteine is involved in the formation of a disulphide bond in EN6 and the tryptophan forms hydrophobic interactions with residues from the neighbouring helix. This may also suggest that despite the structural similarity in the hexapeptide motifs between Peptide 1 and EN6 (Figure 2), their overall interactions govern different biological functions.

### Free energy surface and dihedral PCA

DSSP plots show the presence of stable secondary structure in the hexapeptide region around residues Tyr20 and Cys21. We have previously shown that the amide chemical shift of a particular residue in the locally structured region of disorded peptides is dependent on its own psi torsion and the phi torsion of its subsequent residue [28]. Therefore, we show the reweighted free energy surface of Tyr20 (psi) and Cys21 (phi) for all peptides (Figure 3).

Figure 3A shows the relative free energy surface for Peptide 1 generated after reweighting scaled MD conformations. There is only one minimum in the free energy landscape mapped to the dihedral psi and phi angles between residues Tyr20 and Cys21. The representative conformations from this free energy minimum well have values for the psi angle of Tyr20 and the phi angle of Cys21 of around –42° and –69°, respectively. Furthermore, visualisation of these conformations shows that Tyr18 has a chi1 torsion angle around ~61°, with CH- π interactions between Pro16 and Tyr18, and the occasional presence of the side chains of Tyr20 and Tyr 24 -in close vicinity to one another, as discussed above (Table 1). The free energy surface for Peptide 2 (Figure 3B) reveals that the relative lowest free energy conformations correspond to a similar helical/turn propensity as peptide 1. By contrast, the free energy surface Peptide 3 (Figure 3C) shows that the free energy minimum occupies a much broader but more defined region compared to those observed for Peptides 1 and 2. The values of the psi angle of Tyr15 range from –50° to +50°. The representative conformation shows that the overall energy minimum well shifts to higher values of psi of Tyr15 compared to that in Peptides 1 and 2. A single dominant conformational cluster where the N-terminus is in close contact with C-terminus was identified from the simulation trajectory of Peptide 3, consistent with the analysis of secondary structure (DSSP).

**Figure 4:**
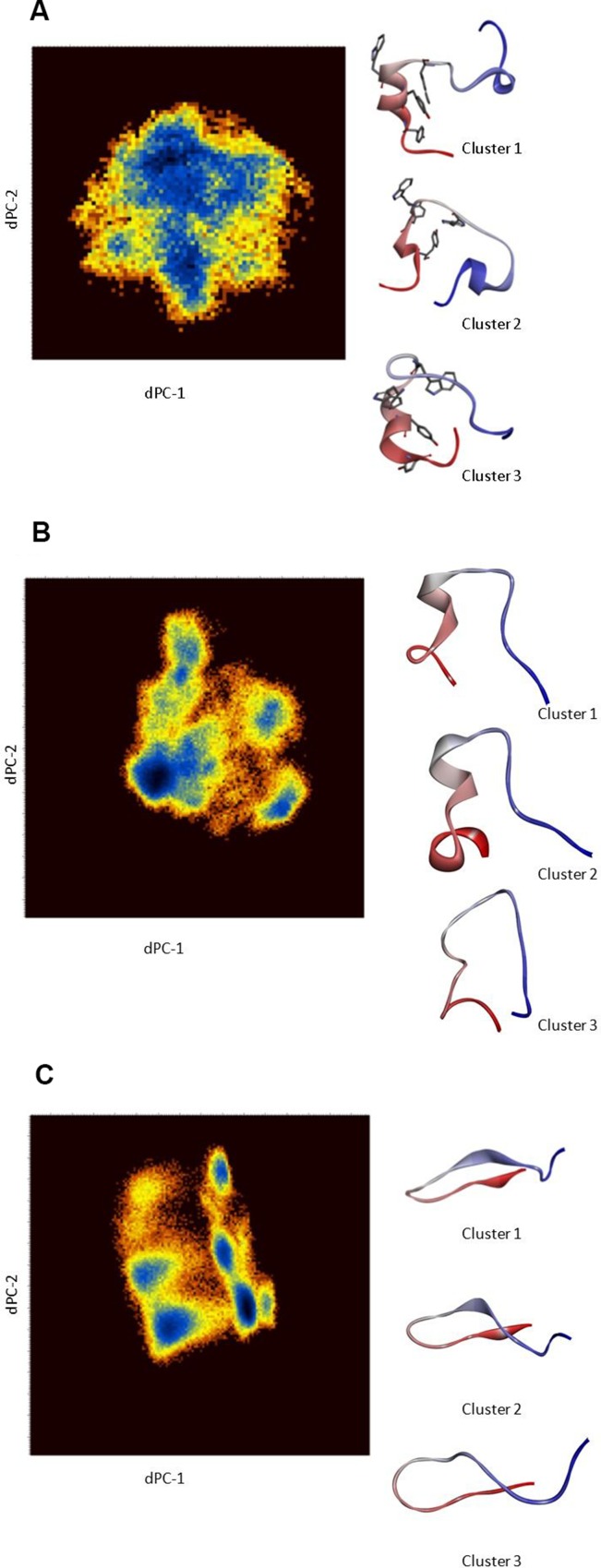
(**A**) Dihedral angle PCA (dPCA) of the backbone and side-chain atoms of Peptide 1 for residues 12–27. The diagrams are pseudo-colour representations of the density functions corresponding to the fluctuations of the N, C′, C atoms in the top two eigenvectors. The representative conformation (coloured from N- to C-terminus) from each dPCA cluster. Clusters 1 and 3 clearly reveal the presence of a helical conformation in the region encompassing the hexapeptide motif. (**B**) Dihedral angle PCA (dPCA) of the backbone and side-chain atoms of Peptide 2 for residues 7–22. The diagrams are pseudo-colour representations of the density functions corresponding to the fluctuations of the N, C′, C atoms in the top two eigenvectors. The representative conformation (coloured from N- to C-terminus) from each dPCA cluster is shown. (**C**) Dihedral angle PCA (dPCA) of the backbone and side-chain atoms of Peptide 3 for residues 7–22. The diagrams are pseudo-colour representations of the density functions corresponding to the fluctuations of the N, C′, C atoms in the top two eigenvectors. The representative conformation (coloured from N- to C- terminal) from each dPCA cluster are shown. These results are from scaled MD simulations with ʎ = 0.7.

Considering that Cartesian coordinates may not be an optimum metric to separate conformations for disordered peptides, a dihedral angle PCA (dPCA) in the sequence PLVWPAWVYCTRYSDR in the EN1-iPeps was conducted to characterise dominant conformational states in more detail (Figure 4). It has been proposed that dPCA can be advantageous for disordered proteins in comparison with classical Cartesian PCA [29, 30]. The free energy surface diagrams are pseudo-colour representations of the density functions (*ΔG = –k_B_* ln(*p/pmax*) corresponding to the fluctuations of the N, C′, Cα atoms in the top two eigenvectors.

In the case of Peptide 1, three conformational states corresponding to the free-energy minima are identified, wherein cluster 1 represents nearly 60% of all of the configurations sampled during the simulation. Representative structures of these conformations are shown in Figure 4A with their average dihedral angles (Supplementary Table 2). Cluster 1 shows the presence of helical conformation in the middle portion of the peptide, whereas the other clusters also indicate the formation of a 3_10_-helix at near the C-terminus. Cluster 1 of Peptides 1 and 2 has a similar dihedral angle distribution (Supplementary Table 2) around the motif ‘W-P-A-W’, indicating the presence of an α-turn conformation (Figure 4A, 4B). By contrast, the representative structures from the dPCA of Peptide 3 (Figure 4C) indicate the presence of beta-hairpin like structures, presumably due to the presence of multiple alanine and ring structures like Tyr15 and Pro11. The presence of beta-strands is also attributed to the presence of a strong ionic interaction between Asp21 from the C-terminus and the positively charged cell penetrating peptide sequence.

Using the PEP-FOLD3 peptide structure prediction approach [31], several viable structural models were generated for Peptides 2 and 3, with model 1 being the best model in most cases. These calculations also predict the existence of probable helices from residues 11 to 16 in Peptide 2, whereas Peptide 3 is predicted to have beta strands with a hairpin fold (Supplementary Figure 3). These predictions are very similar to those predicted by our sMD simulations and are indicative of helical propensity in the hexapeptide motif, except that there are subtle differences in the N- and C- termini of these peptides.

sMD simulation with a scaling factor ʎ = 0.5 also showed the presence of helical conformation in the hexapeptide motif of Peptide 2 (Supplementary Figure 4). The N-terminus was found to be disordered in both sMD simulations with ʎ = 0.5 and 0.7. The free energy surface for Peptide 2 based on residues Tyr15 (psi) and Cys16 (phi) reveals that the relative lowest free energy conformations correspond to a similar helical/turn propensity as observed in the simulation with ʎ = 0.7 (Supplementary Figure 5). It is evident from Supplementary Figure 6 that all the three clusters share the characteristics of an α-helix. A comparison of Ramachandran plots after population reweighting can be found in the Supplementary Information (Supplementary Table 2) for simulations with ʎ = 0.7 and 0.5. Scaled MD simulations with ʎ = 0.5 resulted in only a modest increase in the sampling of the phi–psi conformational space with respect to the simulation with ʎ = 0.7 in the disordered N-terminal region. It has previously been shown that the folding of a protein to its native state can be accelerated within a few hundred nanoseconds using enhanced sampling techniques like accelerated MD/sMD [32], although the success rate is somewhat lower compared to Replica Exchange Molecular Dynamics (REMD). The folding of a protein using sMD is similar to the folding at room temperatures, although sampling of high energy states, and hence more extended conformations, observed at higher temperatures in REMD is less efficient with sMD. Similar convergence issues using sMD (with ʎ = 0.7) has been recently reported for a V3 loop sequence of an R5-tropic HIV-1 strain [33].

The DSSP plot of the sMD simulation of Peptide 3 conducted with ʎ = 0.5 shows some propensity for helical conformation in addition to the structures obtained with ʎ = 0.7 (Supplementary Figure 7). The weighted free energy surface for Peptide 3 (Supplementary Figure 8) and the corresponding Ramachandran plots of residues 7–21 (Supplementary Table 3) suggest that the free energy surface obtained from the sMD simulation with ʎ = 0.7 was not converged and that the hexapeptide conformations were not sufficiently sampled, with visible gaps in the sampling of phi–psi angles. Unweighted clustering analyses indicate the presence of dominant clusters containing ~90% of all conformations sampled with either a hairpin-like fold or random coil. The representative structures from the top three clusters are shown in Supplementary Figure 9. The DSSP plot during the last 40 ns of the simulation of Peptide 3 with ʎ = 0.5 is similar to that for the simulation of Peptide 2 (Supplementary Figures 4 and 7). Structures extracted from the last 40 ns of the simulations of both peptides superimpose well with an RMSD < 2.5 Å in the hexapeptide motifs. Contact residue analyses of the side-chain atoms from the last 40 ns of the simulations of both peptides reveal the importance of aromatic residues (Supplementary Figure 10). In Peptide 2, Tyr15 is within a cutoff distance of 6 Å to Trp10, whereas the same Tyr15 in Peptide 3 forms a cation-π interaction with Arg18. The hydrogen bonds in Peptide 2 between the backbone pairs Trp13-Tyr15, Ala12-Cys16, Trp13-Thr17 and Trp13-Tyr19 were observed 25%, 15%, 16% and 36% of the time (during the last 40 ns), respectively. These results are in agreement with previously reported simulations and the crystal structure of HOX hexapeptides, wherein a strong tendency to form backbone hydrogen bonds between the carbonyl oxygen of residue i and the amide hydrogen of residue i + 3/i + 4 [14] is observed. The hydrogen bonds between the backbone of Val14-Cys16 and Pro11-Tyr14/Val14 most probably supports a folded conformation in Peptide 3. These results suggest that hexapeptide motifs in EN1-iPeps are able to remain folded to some extent even without aromatic side chains; however, inter-residue interactions are different between the WT (peptide 1 and 2) and mutated (peptide 3) peptides.

Apart from CH-π interactions, the role of two tryptophan residues in the EH2 domain has been implicated in cooperative binding of En2 with Pbx and Hox gene products [5]. Mutation of tryptophan to arginine leads to a complete loss of formation of En2- Pbx complexes. We provide an explanation below of the role of aromatic residues in the binding to their partners.

### Structural conservation between the EN1-iPep and HOX members

**Figure 5:**
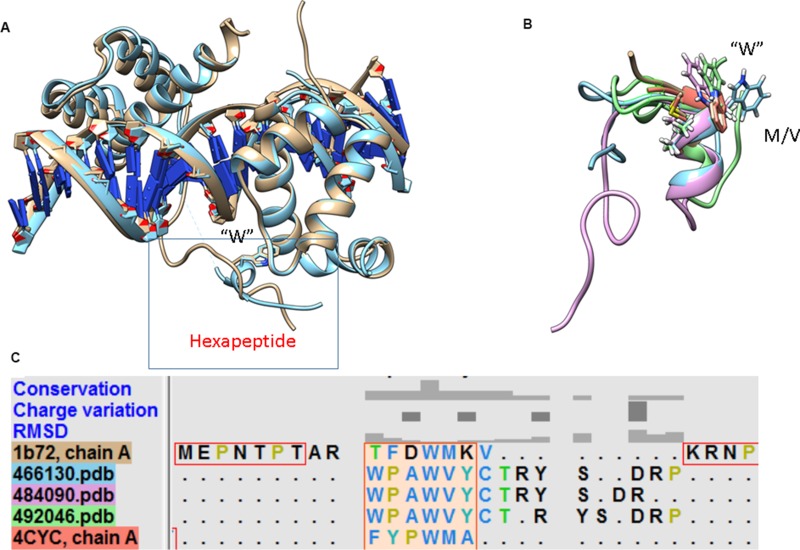
Structural superimposition of members from the anterior and posterior HOX family in complex with DNA and partner proteins (**A**) Despite structural differences in the hexapeptide motif, the position of tryptophan in the hydrophobic pocket is conserved. (**B**) The structural alignment of the hexapeptide motif in the EN1-iPep (Peptide 1) and anterior HOX members reveals that tryptophan is conserved. (**C**) The presence of “CTRY” in EN1 facilitates the formation of a longer helix, giving rise to binding specificity.

The 3D structures of the hexapeptide motif in posterior and anterior HOX proteins are strikingly different, with the exception of the identical position of the conserved tryptophan residue within the hexapeptide-binding pocket of Pbx. In the case of the anterior HOXB1-Pbx1-DNA complex [12] and the central posterior Ultrabithorax (Ubx)-EXB-DNA complex [11], the Hox protein in each structure interacts with the PBC protein using its hexapeptide motif, which has the sequence TFDWM in the case of HoxB1 and FYPWM in the case of Ubx. In both cases, the hexapeptide motif adopts a 3_10_-helix conformation that packs against the PBC homeodomain and inserts the conserved tryptophan into the hexapeptide-binding pocket. We superimposed the structure of posterior HoxA9 complexed with Pbx1 and DNA (PDB code 1PUF), which revealed that the hexapeptide motif has a very divergent sequence and adopts a disordered conformation compared to that in the HOX paralogue members 1 to 8. Figure 5 shows that the position of the invariant tryptophan is nonetheless highly conserved. Based on this observation, a structural alignment of the low energy/most populated conformations of Peptide 1 with the anterior HOX 3D structures (PDB codes 1B72 and 4CYC) was done using the MatchMaker algorithm in UCSF chimera (Figure 5A). The structural alignment reveals the highly conserved nature of W18 in the helix (Figure 5B), despite the different sequence of the hexapeptide motif (Figure 5C), suggesting that the presence of tryptophan may be the principal determinant for binding in all HOX members to the pocket found in their partner proteins.

## MATERIALS AND METHODS

MD simulations of EN1-iPeps were performed using the AMBER simulation package version 14 [34]. Energy minimisation was done using the parallel CPU implementation of AMBER pmemd and all subsequent simulations used the parallel GPU implementation of AMBER pmemd [35]. The unfolded initial structures of EN1-iPeps (Table 4) were constructed using the leap program in AmberTools15. It should be noted that the trans conformation of proline residues is always the dominating isomer in short peptides in water, and in Hox family members the contribution of *cis* isomers is negligible [15]. Therefore, all proline residues were modelled in the *trans* conformation in all simulations. The peptides were energy minimised and subjected to a 50 ps simulation in implicit solvent. The EN1-iPeps were then placed in a truncated octahedral box of pre-equilibrated TIP3P water such that the minimum distance between any peptide atom and the edge of the box is at least 15 Å. The positive charge in the peptides was neutralised by adding chloride ions. Sodium and chloride ions were also added to represent an ionic strength of 50 mM. The resulting simulation conditions of the various EN1-iPeps are summarised in Table 3. The peptide interactions were modelled with the Amber 14SB force field [36], the ions were modelled with the parameters from Joung and Cheatham [37] and water with the TIP3P potential [38].

**Table 4:** List of systems simulated

EN1-iPep sequence	Experimental IC_50_	Number of ions	Number of water molecules
^1^KKKRKVTDSQQPLV^15^WPAWVY^20^CTRYSDRPS^29^	17.5 μM [3]	33	6846
^1^KKKRKVPLV^10^WPAWVY^15^CTRYSDR^22^	9.28 μM [4]	23	4481
^1^KKKRKVPLV^10^APAAVY^15^CTRYSDR^22^	>50 μM [4]	29	5742

The cell penetrating sequence is shown in red and the conserved hexapeptide motif is shown in green.

The solvated EN1-iPep systems were equilibrated using a four stage procedure: energy minimisation, heating with restraints, heating removing restraints, and unrestrained dynamics. Energy minimisation was carried out with 1000 steps of the steepest descent algorithm and 4000 steps of conjugate gradients. For simulations of the iPeps in water, heating was carried out in two stages. The first stage was performed in the NVT ensemble, and the temperature was increased from 0 to 100 K over 250 ps while imposing harmonic restraints on the backbone of the protein with a force constant of 10.0 kcal mol^−1^ Å^−2^. In the second stage the temperature was increased from 100 to 298 K over 250 ps in the NVT ensemble. Once the system reached its final temperature, the backbone restraints and a 500 ps simulation in the NPT ensemble was conducted to ensure that the systems had reached the appropriate density. The integration time step for all simulations was 2 fs. The particle mesh Ewald (PME) method [39] was used to compute long-range electrostatic interactions, with a cutoff of 12 Å for all non-bonded interactions. Temperature control was implemented using the Langevin thermostat method with a collision frequency of 2 ps^−1^ [40]. The SHAKE algorithm was employed to restrain hydrogen atoms [41]. Weak coupling (Berendsen) to an external pressure bath was used to control the pressure [42]. Subsequent to the equilibration, simulations were continued at 298 K using the sMD method [22] in order to enhance sampling of protein conformational transitions. The scaling parameter lambda (ʎ) was set to 0.7 and simulations were performed in the NPT ensemble for 500 ns. To check for convergence, a second sMD simulations for Peptides 2 and 3 were carried out using a new starting structure, initial velocities and with scaling parameter ʎ = 0.5, for 500 ns. Lower values of ʎ can greatly enhance sampling of high energy states, including rotations of omega torsions [22]. In order to prevent cis-trans isomerization of peptide bonds and maintain a balance between enhanced sampling and a physically relevant conformational ensemble, sMD simulations should be performed with ʎ > 0.4. Population-based reweighting was carried out using the Python script provided by the developers of sMD on their website. Discretisation of 1, phi and psi torsions and ʎ values of 0.7 or 0.5 were used for reweighting analysis.

Trajectories were saved every 5 ps for post-processing analysis. Analyses of secondary structure, hydrogen bonds and other interactions were performed using the program cpptraj in AmberTools 15. Distance and angle cut-offs of <3.5 Å and <35°, respectively, were used for computing hydrogen bonds. A cut-off distance of 6 Å from sidechain atoms only was used for calculating residue contacts. Analysis and visualisation was done using UCSF Chimera [43]. Dihedral principal component analysis (dPCA) was performed using Carma [44] on 100,000 frames from each trajectory. In the dPCA technique, dihedral angles are transformed to the metric coordinate space: x(n) = cos phi(n) and y(n) = sin phi(n), where n is the number of dihedral angles used in the analysis. A peak-picking algorithm for cluster analysis was applied to the three-dimensional density distributions of the principal components derived from the MD trajectory. The phi, psi and chi1 torsion angles were all considered. Clustering in CARMA is aimed at identifying prominent molecular configurations but cannot be used to determine transitions and average lifetimes of conformations. The same configurations were used to predict backbone and side-chain chemical shifts using the SHIFTX2 program [45]. All the above analyses, except the phi-psi free energy maps, were performed on the biased simulations.

The dominant structures of the EN1-iPeps predicted by the MD simulations were compared with predictions from *de novo* structure approaches. For this purpose, the sequences of Peptides 2 and 3 were submitted to the PEP-FOLD3 server [31], which uses a coarse grained approach coupled to a greedy algorithm to predict peptide structure in solution. The server also performs clustering using the sOPEP force field.

## CONCLUSIONS

A truncated form of the Engrailed-1 protein, which includes a hexapeptide motif along with a cell penetrating sequence (iPep) was studied using molecular dynamics simulations. The free energy landscape and chemical shift analyses reveal that the conserved tryptophan-containing hexapeptide motif in the EN1-iPep adopts a stable helical conformation in solution despite the fact that it is a truncated peptide from the full-length transcription factor. This is similar to what was previously determined for anterior HOX/PBX structures (i.e. like HoxA9/Pbx1/DNA). Deletion of non-conserved residues in EN1-iPep (Peptide 2) results in a more compact structure with a helical conformation followed by turn/bends on both termini. When aromatic residues (W) are changed to alanine, the folding of the EN1-iPeps from the extended peptide conformation were hindered. Simulations of Peptides 1 and 2 suggest that aromatic side chains are important in the folding processes due to the presence of intramolecular hydrogen bonds, π - π and CH- π interactions. Furthermore, the loss of helical structure upon mutation of tryptophan (Peptide 3) indicates that this residue is indispensable for the anti-cancer activity of these EN1-iPeps. The cell penetrating sequence in all the three peptides was found to be disordered, with several transient conformations. The flanking residues of the dipeptide -W-M/A might govern the specificity of binding between Hox and EN proteins.

This study shows to the benefit of complementing MD simulations with NMR data in order to obtain a more comprehensive picture at the atomistic level of the conformational dynamics taking place in the nanosecond timescale. The chemical shifts of Peptides 1 and 2 correlate well with those of HOX hexapeptides in solution, with the NMR structures of En6 and En2 indicating the presence of turn/helical structures. Our observations imply that the hexapeptide motif is at least partially ′preformed' in the EN1-iPep and thus ready to interact with its binding partners. Therefore, the design and synthesis of analogues that mimic the helical turn conformation and, in particular, the presence of aromatic hydrophobic residue like tryptophan in the Hox hexapeptide motif may have potentially chemotherapeutic properties against cancers by virtue of inhibiting the interaction of EN1 with its partner proteins and DNA.

## SUPPLEMENTARY MATERIALS FIGURES AND TABLES







## References

[1] Shah N, Sukumar S (2010). The Hox genes and their roles in oncogenesis. Nat Rev Cancer.

[2] Zec N, Rowitch DH, Bitgood MJ, Kinney HC (1997). Expression of the homeobox-containing genes EN1 and EN2 in human fetal midgestational medulla and cerebellum. J Neuropathol Exp Neurol.

[3] Beltran AS, Graves LM, Blancafort P (2014). Novel role of Engrailed 1 as a prosurvival transcription factor in basal-like breast cancer and engineering of interference peptides block its oncogenic function. Oncogene.

[4] Sorolla A, Ho D, Wang E, Evans CW, Ormonde CF, Rashwan R, Singh R, Iyer KS, Blancafort P (2016). Sensitizing basal-like breast cancer to chemotherapy using nanoparticles conjugated with interference peptide. Nanoscale.

[5] Peltenburg LT, Murre C (1996). Engrailed and Hox homeodomain proteins contain a related Pbx interaction motif that recognizes a common structure present in Pbx. EMBO J.

[6] Morris MC, Deshayes S, Heitz F, Divita G (2008). Cell-penetrating peptides: from molecular mechanisms to therapeutics. Biol Cell.

[7] Plowright L, Harrington KJ, Pandha HS, Morgan R (2009). HOX transcription factors are potential therapeutic targets in non-small-cell lung cancer (targeting HOX genes in lung cancer). Br J Cancer.

[8] Lu Q, Kamps MP (1996). Structural determinants within Pbx1 that mediate cooperative DNA binding with pentapeptide-containing Hox proteins: proposal for a model of a Pbx1-Hox-DNA complex. Mol Cell Biol.

[9] Sprules T, Green N, Featherstone M, Gehring K (2003). Lock and Key Binding of the HOX YPWM Peptide to the PBX Homeodomain. J Biol Chem.

[10] Knoepfler PS, Kamps MP (1995). The pentapeptide motif of Hox proteins is required for cooperative DNA binding with Pbx1, physically contacts Pbx1, and enhances DNA binding by Pbx1. Mol Cell Biol.

[11] Passner JM, Ryoo HD, Shen L, Mann RS, Aggarwal AK (1999). Structure of a DNA-bound Ultrabithorax-Extradenticle homeodomain complex. Nature.

[12] Piper DE, Batchelor AH, Chang CP, Cleary ML, Wolberger C (1999). Structure of a HoxB1-Pbx1 heterodimer bound to DNA: role of the hexapeptide and a fourth homeodomain helix in complex formation. Cell.

[13] Slupsky CM, Sykes DB, Gay GL, Sykes BD (2001). The HoxB1 hexapeptide is a prefolded domain: Implications for the Pbx1/Hox interaction. Protein Sci.

[14] Rundgren H, Mark P, Laaksonen A (2007). Molecular dynamics simulations of conserved Hox protein hexapeptides. I. Folding behavior in water solution. J Mol Struct THEOCHEM.

[15] Rundgren H, Mark P, Laaksonen A (2007). Molecular dynamics simulations of conserved Hox protein hexapeptides II. Folded structures in water solution. J Mol Struct THEOCHEM.

[16] Koulgi S, Sonavane U, Joshi R (2010). Insights into the folding pathway of the Engrailed Homeodomain protein using replica exchange molecular dynamics simulations. J Mol Graph Model.

[17] McCully ME, Beck DA, Daggett V (2008). Microscopic reversibility of protein folding in molecular dynamics simulations of the engrailed homeodomain. Biochemistry.

[18] Clarke ND, Kissinger CR, Desjarlais J, Gilliland GL, Pabo CO (1994). Structural studies of the engrailed homeodomain. Protein Sci.

[19] Zhao X, Huang XR, Sun CC (2006). Molecular dynamics analysis of the engrailed homeodomain–DNA recognition. J Struct Biol.

[20] Religa TL (2008). Comparison of multiple crystal structures with NMR data for engrailed homeodomain. J Biomol NMR.

[21] Logan C, Hanks MC, Noble-Topham S, Nallainathan D, Provart NJ, Joyner AL (1992). Cloning and sequence comparison of the mouse, human, and chicken engrailed genes reveal potential functional domains and regulatory regions. Dev Genet.

[22] Sinko W, Miao Y, de Oliveira CA, McCammon JA (2013). Population based reweighting of scaled molecular dynamics. J Phys Chem B.

[23] Tóth G, Murphy RF, Lovas S (2001). Stabilization of local structures by π-CH and aromatic-backbone amide interactions involving prolyl and aromatic residues. Protein Eng.

[24] Dujardin M, Madan V, Montserret R, Ahuja P, Huvent I, Launay H, Leroy A, Bartenschlager R, Penin F, Lippens G, Hanoulle X (2015). A Proline-Tryptophan Turn in the Intrinsically Disordered Domain 2 of NS5A Protein is Essential for Hepatitis C Virus RNA Replication. J Biol Chem.

[25] Augustyniak R, Balayssac S, Ferrage F, Bodenhausen G, Lequin O (2011). 1H, 13C and 15N resonance assignment of a 114-residue fragment of Engrailed 2 homeoprotein, a partially disordered protein. Biomol NMR Assign.

[26] Jung S, Dingley AJ, Augustin R, Anton-Erxleben F, Stanisak M, Gelhaus C, Gutsmann T, Hammer MU, Podschun R, Bonvin AM, Leippe M, Bosch TC, Grotzinger J (2009). Hydramacin-1, structure and antibacterial activity of a protein from the basal metazoan Hydra. J Biol Chem.

[27] Pedrini B, Placzek WJ, Koculi E, Alimenti C, LaTerza A, Luporini P, Wuthrich K (2007). Cold-adaptation in sea-water-borne signal proteins: sequence and NMR structure of the pheromone En-6 from the Antarctic ciliate Euplotes nobilii. J Mol Biol.

[28] Gandhi NS, Landrieu I, Byrne C, Kukic P, Amniai L, Cantrelle FX, Wieruszeski JM, Mancera RL, Jacquot Y, Lippens G (2015). A Phosphorylation-Induced Turn Defines the Alzheimer's Disease AT8 Antibody Epitope on the Tau Protein. Angew Chem Int Ed Engl.

[29] Espinoza-Fonseca LM, Ilizaliturri-Flores I, Correa-Basurto J (2012). Backbone conformational preferences of an intrinsically disordered protein in solution. Mol Biosyst.

[30] Altis A, Nguyen PH, Hegger R, Stock G (2007). Dihedral angle principal component analysis of molecular dynamics simulations. J Chem Phys.

[31] Lamiable A, Thévenet P, Rey J, Vavrusa M, Derreumaux P, Tufféry P (2016). PEP-FOLD3: faster de novo structure prediction for linear peptides in solution and in complex. Nucleic Acids Res.

[32] Doshi U, Hamelberg D (2015). Towards fast, rigorous and efficient conformational sampling of biomolecules: advances in accelerated molecular dynamics. Biochim Biophys Acta.

[33] Nemec M, Hoffmann D (2017). Quantitative Assessment of Molecular Dynamics Sampling for Flexible Systems. J Chem Theory Comput.

[34] Case DA, Babin V, Berryman JT, Betz RM, Cai Q, Cerutti DS, Cheatham ITE, Darden TA, Duke RE, Gohlke H, Goetz AW, Gusarov S, Homeyer N (2014). AMBER.

[35] Salomon-Ferrer R, Case DA, Walker RC (2013). An overview of the Amber biomolecular simulation package. Wiley Interdiscip Rev Comput Mol Sci.

[36] Maier JA, Martinez C, Kasavajhala K, Wickstrom L, Hauser KE, Simmerling C (2015). ff14SB: Improving the Accuracy of Protein Side Chain and Backbone Parameters from ff99SB. J Chem Theory Comput.

[37] Joung IS, Cheatham TE (2008). Determination of alkali and halide monovalent ion parameters for use in explicitly solvated biomolecular simulations. J Phys Chem B.

[38] Jorgensen WL, Chandrasekhar J, Madura JD, Impey RW, Klein ML (1983). Comparison of simple potential functions for simulating liquid water. J Chem Phys.

[39] Darden T, York D, Pedersen L (1993). Particle mesh Ewald: An N·log(N) method for Ewald sums in large systems. J Chem Phys.

[40] Loncharich RJ, Brooks BR, Pastor RW (1992). Langevin dynamics of peptides: the frictional dependence of isomerization rates of N-acetylalanyl-N’-methylamide. Biopolymers.

[41] Ryckaert JP, Ciccotti G, Berendsen HJ (1977). Numerical integration of the cartesian equations of motion of a system with constraints: molecular dynamics of n-alkanes. J Comput Phys.

[42] Berendsen HJ, Postma JV, van Gunsteren WF, DiNola A, Haak J (1984). Molecular dynamics with coupling to an external bath. J Chem Phys.

[43] Pettersen EF, Goddard TD, Huang CC, Couch GS, Greenblatt DM, Meng EC, Ferrin TE (2004). UCSF Chimera—a visualization system for exploratory research and analysis. J Comput Chem.

[44] Glykos NM (2006). Software news and updates carma: A molecular dynamics analysis program. J Comput Chem.

[45] Han B, Liu Y, Ginzinger SW, Wishart DS (2011). SHIFTX2: significantly improved protein chemical shift prediction. J Biomol NMR.

