# Supplementary material for: Atomistic molecular dynamics simulations of bioactive engrailed 1 interference peptides (EN1-iPeps)
Source: Oncotarget. 2018 Apr 27;9(32):22383–97. doi: 10.18632/oncotarget.25025 (PMC5976472; doi:10.18632/oncotarget.25025)
Supplement: Supplementary file 1 [file oncotarget-09-22383-s001.pdf]

Atomistic molecular dynamics simulations of bioactive engrailed 1 interference peptides (EN1-iPeps)

SUPPLEMENTARY MATERIALS

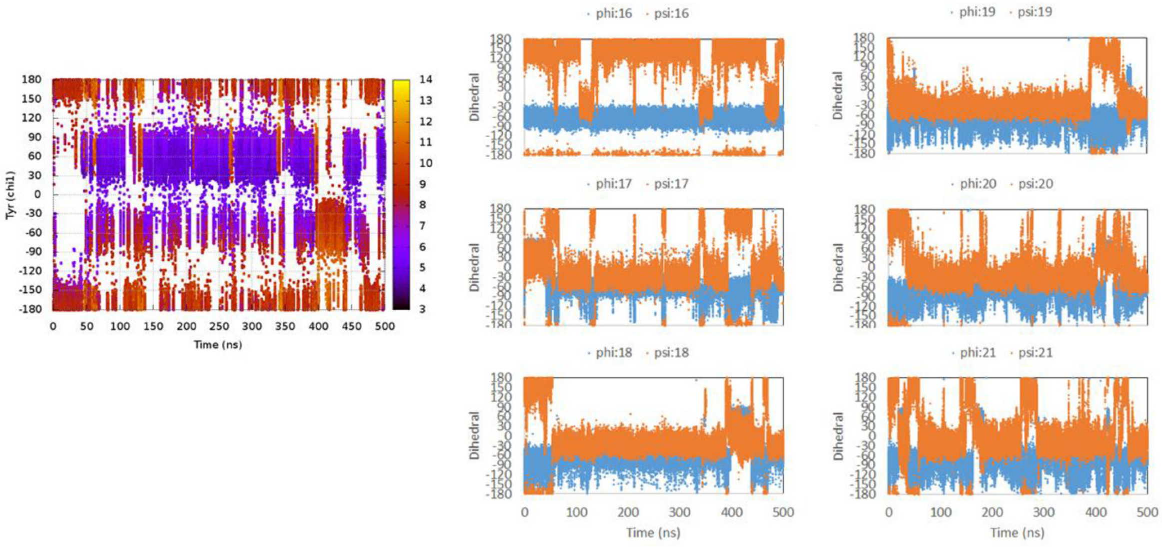

Supplementary Figure 1: Time evolution of the chi1 torsion angle of Tyr18, the distance between Pro16 and Tyr18 (indicated by the colour bar) and the dihedral torsion angles of residues "PAWVYC" in Peptide 1 (sMD λ = 0.7). When the value of chi1 of Tyr18 is ~61°, the distance between the sidechains of Pro16 and Tyr18 is ~5 Å (highlighted in purple), indicating the presence of CH-π interactions. Simultaneously, the negative values of the dihedral angle torsions correspond to helical conformations. This suggests a role for these side chains in stabilizing the helical conformation in the hexapeptide region.

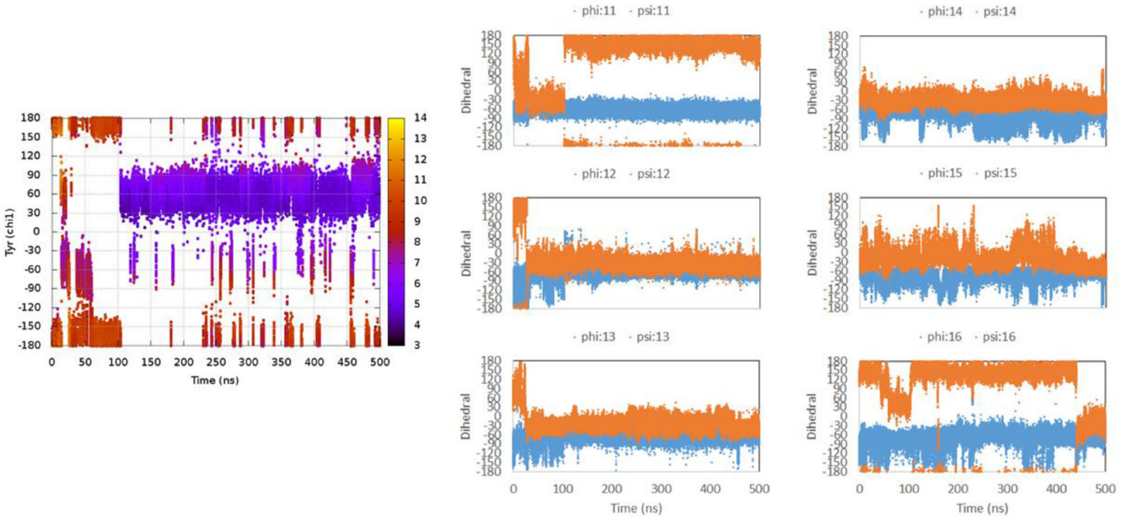

Supplementary Figure 2: Time evolution of the distance between Pro11 and Tyr13 (indicated by the colour bar), the chi1 torsion angle of Tyr13 and the dihedral torsion angles of residues "PAWVYC" in Peptide 2 (sMD ʎ = 0.7). When the value of chi1 of Tyr13 is ~61°, the distance between the side chains of Pro11 and Tyr13 is ~5 Å (highlighted in purple), indicating the presence of CH-π interactions. Simultaneously, the negative values of dihedral angle torsions correspond to helical conformations. This suggests a role for these side chains in stabilizing the helical conformation in the hexapeptide region.

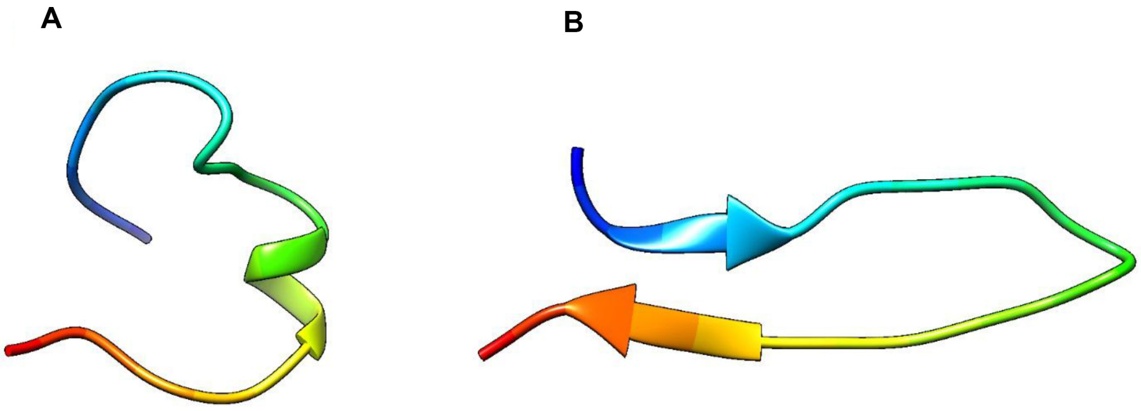

Supplementary Figure 3: PEP-FOLD3 server-generated conformations for (**A**) Peptide 2 and (**B**) Peptide 3, obtained from initial extended conformations. The structures are shown in a ribbon cartoon representation and coloured from the N- to the C-terminus.

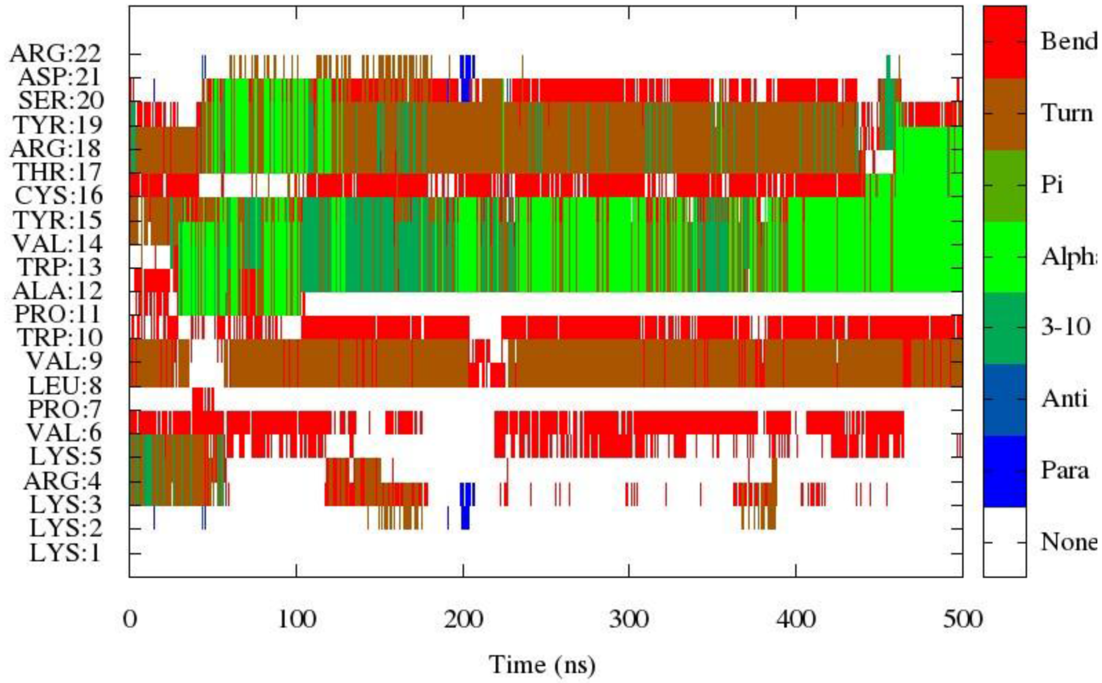

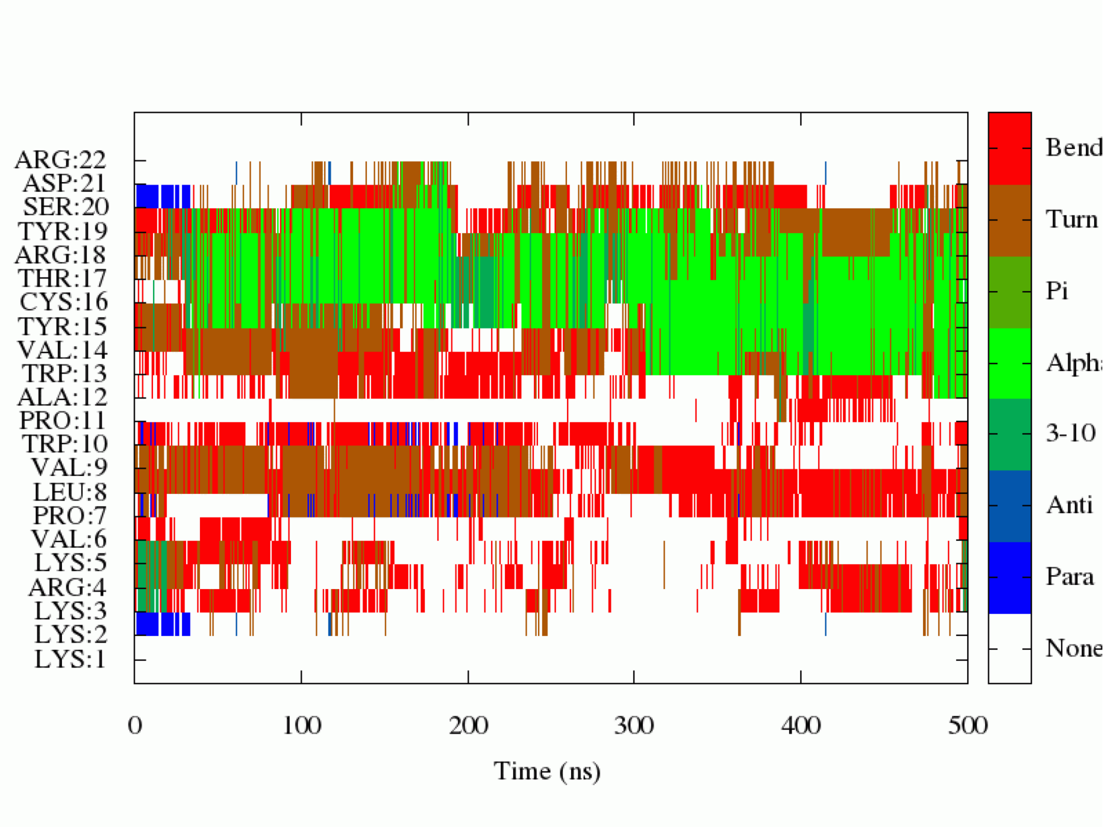

Supplementary Figure 4: Evolution of the secondary structure of Peptide 2 in the sMD simulations with λ = 0.7 and 0.5, respectively.

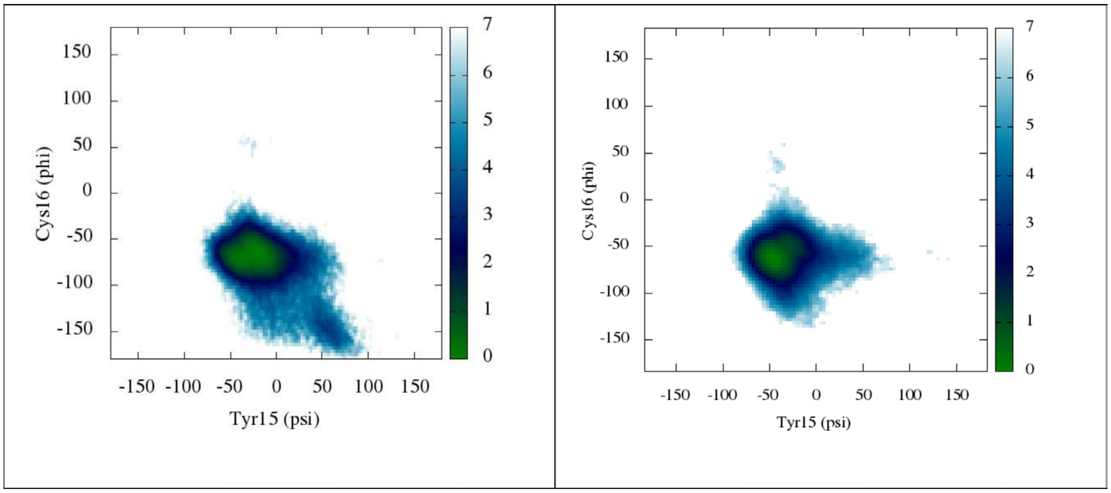

Supplementary Figure 5: Backbone torsional free energy landscapes of Peptide 2 in the SMD simulations with λ = 0.7 and 0.5, respectively.

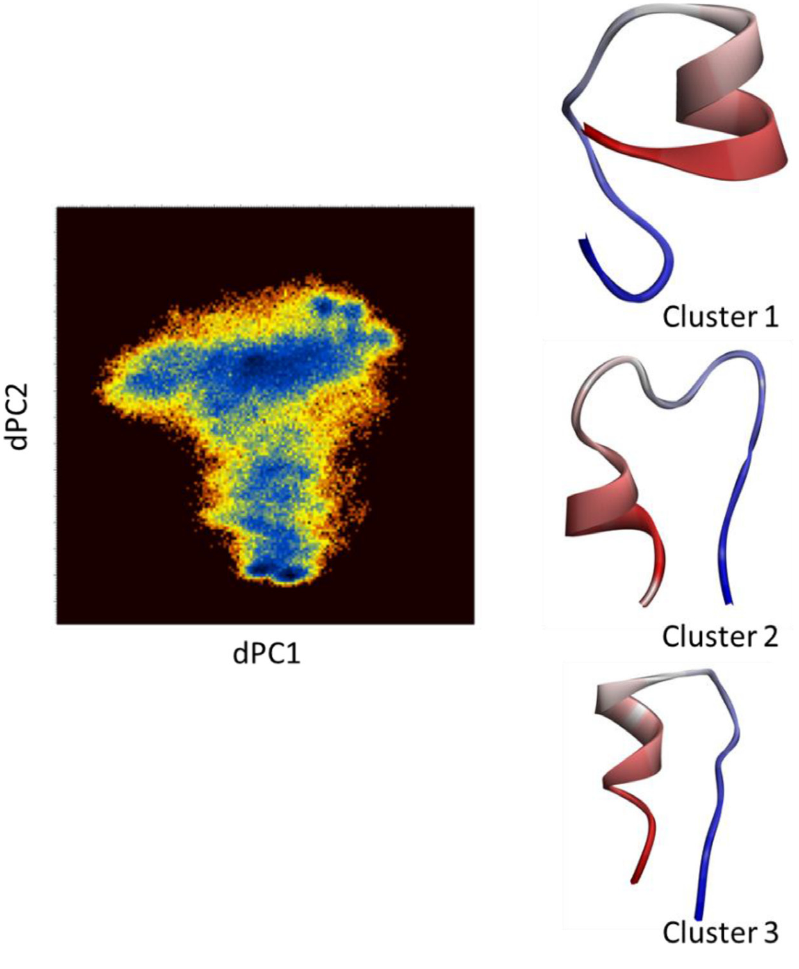

Supplementary Figure 6: Dihedral angle PCA (dPCA) of the backbone and sidechain atoms of Peptide 2 for residues 7–22 in the sMD simulation with λ = 0.5. The diagrams are pseudo-colour representations of the density functions corresponding to the fluctuations of the N, C′, C atoms in the top two eigenvectors. The representative conformation (coloured from N- to C-terminus) from each dPCA cluster is shown.

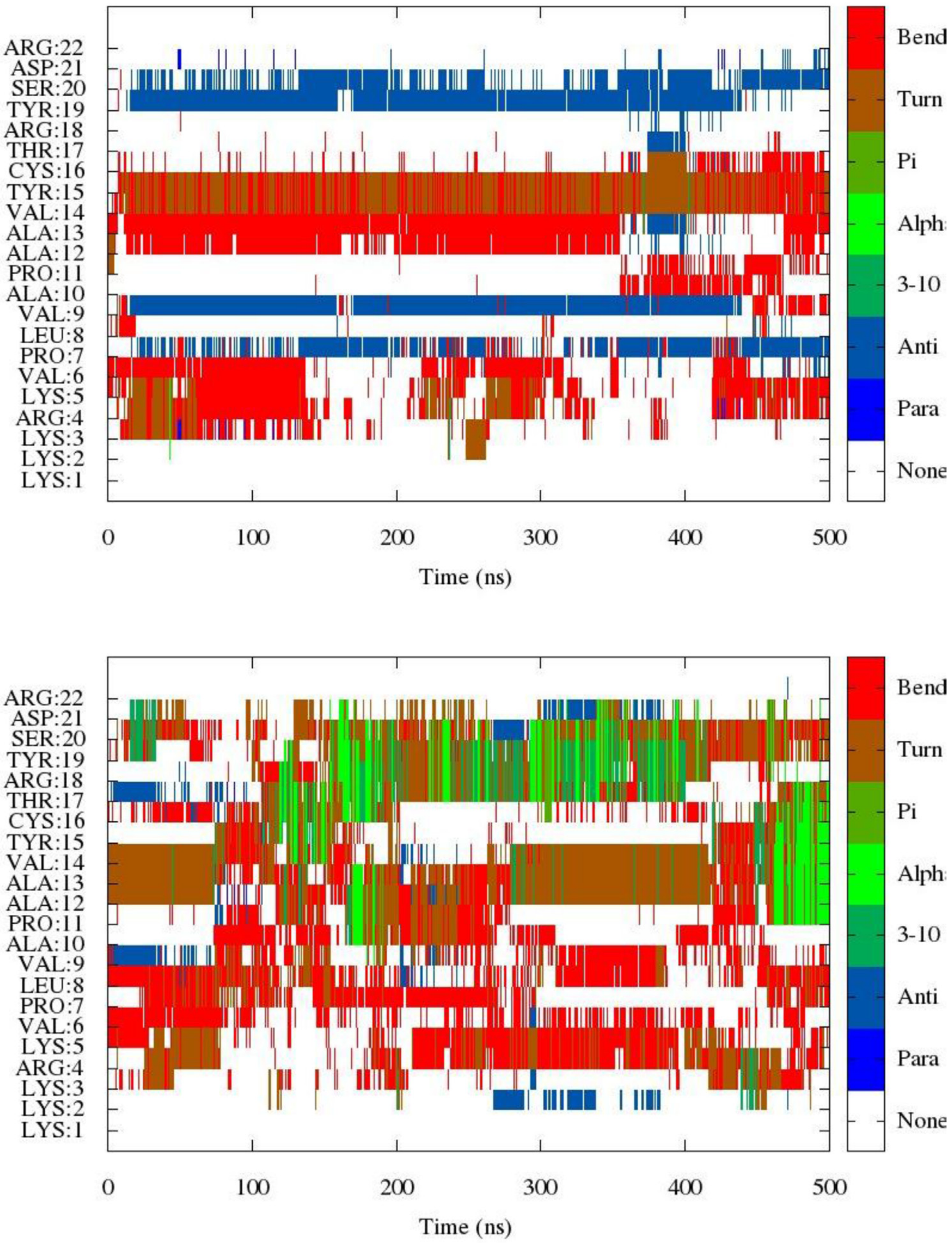

Supplementary Figure 7: Evolution of the secondary structure of Peptide 3 in the SMD simulations with ƙ = 0.7 and 0.5, respectively.

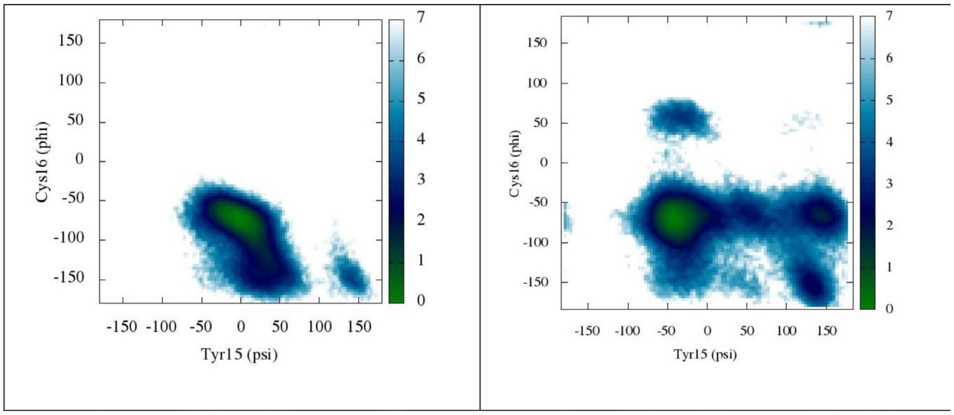

Supplementary Figure 8: Backbone torsional free energy landscapes of Peptide 3 in the SMD simulations with λ = 0.7 and 0.5, respectively.

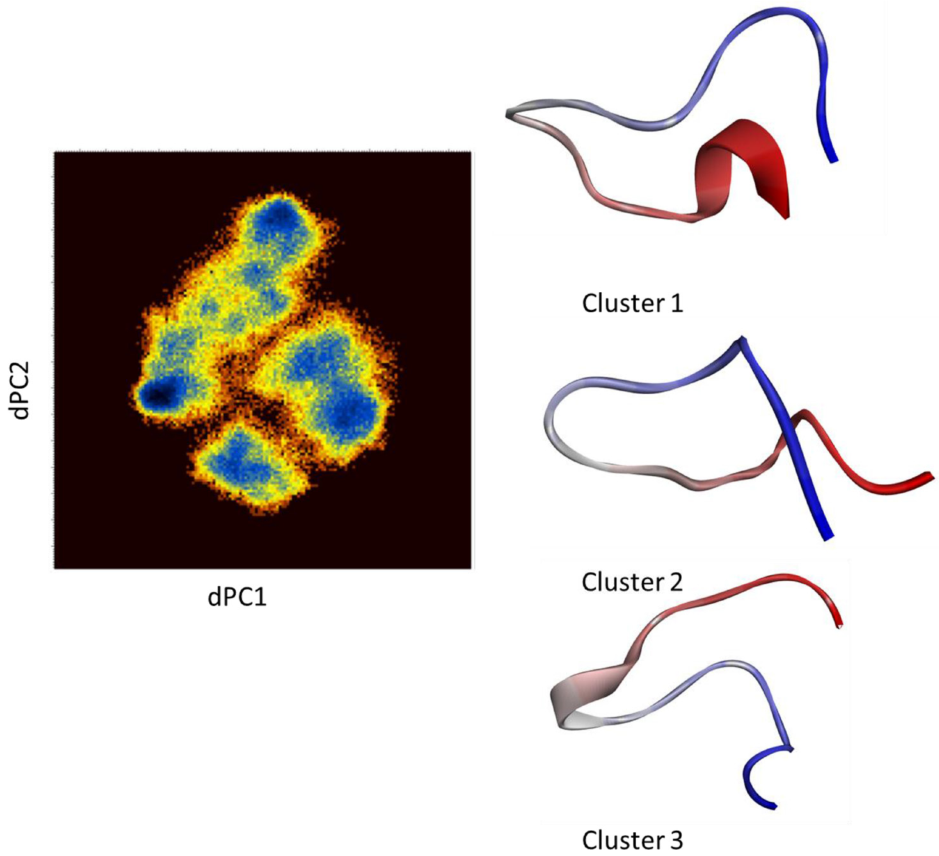

Supplementary Figure 9: Dihedral angle PCA (dPCA) of the backbone and side-chain atoms of peptide 3 for residues 7–22 from scaled MD simulation with λ = 0.5. The diagrams are pseudo-colour representations of the density functions corresponding to the fluctuations of the N, C′, C atoms in the top two eigenvectors. The representative conformation (coloured from N- to C-terminus) from each dPCA cluster is shown.

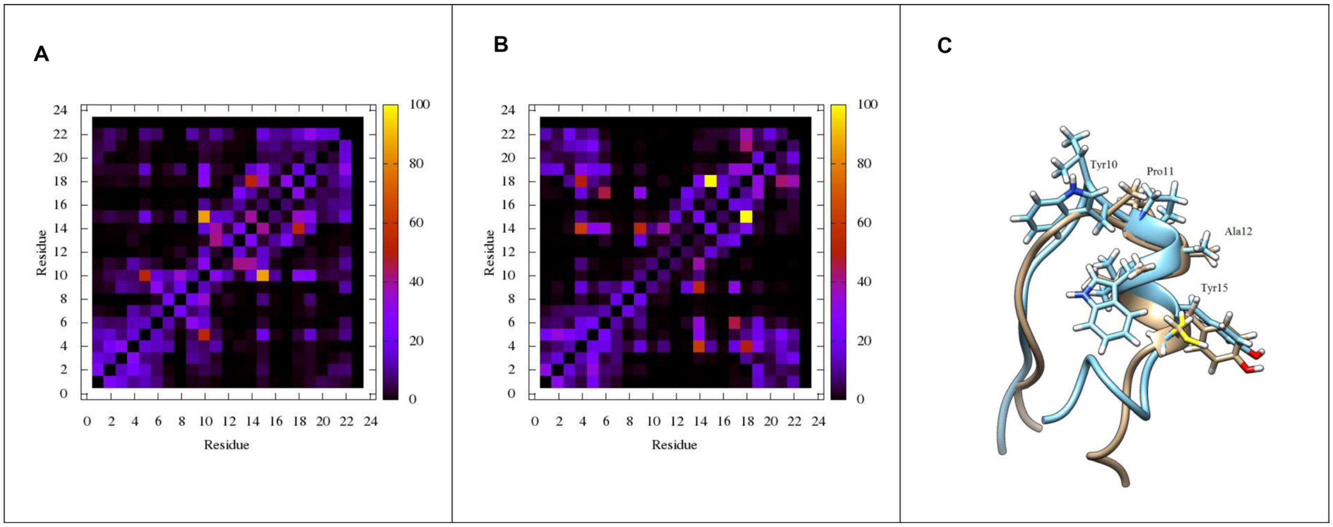

Supplementary Figure 10: Contact map for residues of (**A**) Peptide 2 and (**B**) Peptide 3 during the last 40 ns of the SMD simulations with ʎ = 0.5. The contact map was calculated using only the sidechain atoms of all residues. The last 40 ns of the SMD simulations show the presence of helical propensity; however, the underlying interactions are different. The contact map indicates the interaction of Tyr15 with Trp10 in Peptide 2, whereas Tyr15 is in close contact with Arg18 in Peptide 3. Superimposition of representative snapshots of helical conformations of both iPeps (**C**) clearly indicates the absence of aromatic interactions in the helical conformations of Peptide 3 (shown in golden colour) compared to Peptide 2 (shown in blue).

Supplementary Table 1: Average dihedral angles in each dPCA cluster in Peptides 1, 2 and 3 derived from SMD simulations with $\kappa = 0.7$

Peptide 1	Cluster 1				Cluster 2				Cluster 3			
Residue	Phi	Psi	SD (Phi)	SD (Psi)	Phi	Psi	SD (Phi)	SD (Psi)	Phi	Psi	SD (Phi)	SD (Psi)
12	–66.15	135.23	12.35	78.66	–67.24	129.31	12.22	61.99	–59.26	134.64	11.56	34.26
13	–81.10	38.41	49.92	77.71	–85.65	88.18	31.45	72.48	57.68	–23.13	34.70	25.47
14	–90.06	–5.02	61.34	84.86	–85.90	98.15	29.94	85.73	–91.41	–27.19	25.63	24.63
15	–91.76	147.46	32.48	19.10	–107.17	143.92	33.17	14.38	–70.63	132.77	24.06	12.21
16	–63.99	146.15	11.35	54.30	–65.81	152.58	11.91	17.60	–64.82	–19.61	10.75	31.17
17	–60.10	–28.62	25.54	36.23	–72.62	147.68	18.91	19.32	–97.62	20.76	31.61	34.77
18	–69.79	–25.01	17.73	39.08	60.77	2.44	11.67	28.22	–64.97	–30.53	18.86	52.66
19	–74.92	–34.14	21.76	26.21	–88.62	133.85	29.66	22.74	–54.28	–33.49	36.92	15.92
20	–75.36	–27.09	25.01	53.75	–37.08	42.47	64.94	46.92	–75.65	–37.14	16.49	24.25
21	–75.37	–12.36	38.56	64.55	–70.91	–18.64	43.05	49.16	–70.24	–23.72	22.41	22.51
22	–85.77	–9.53	44.76	81.38	–66.79	–19.70	21.30	16.31	–71.81	–26.68	20.99	20.00
23	–83.76	9.95	61.27	79.30	–73.99	–25.04	20.80	18.74	–86.02	–31.78	25.32	18.64
24	–89.78	58.50	46.39	79.61	–118.31	1.25	25.13	56.75	–109.16	31.61	27.48	48.02
25	–91.08	56.20	63.89	81.63	–107.15	153.63	37.43	16.06	–79.28	–5.43	49.26	51.55
26	–88.39	11.71	47.81	81.52	–77.04	–6.74	41.40	49.93	–86.88	–10.80	26.72	30.21
27	–95.81	140.83	44.50	53.13	–112.69	144.92	37.50	22.02	–124.63	145.39	33.85	24.95

Peptide 2	Cluster 1				Cluster 2				Cluster 3			
Residue	Phi	Psi	SD (Phi)	SD(Psi)	Phi	Psi	SD (Phi)	SD (Psi)	Phi	Psi	SD (Phi)	SD (Psi)
7	–65.43	148.79	13.20	15.55	–67.93	139.98	10.80	16.45	–63.97	148.74	10.62	18.23
8	–64.47	–19.73	13.64	21.54	–66.23	–8.76	12.32	19.75	–65.02	–12.41	11.54	22.46
9	–73.41	–16.51	23.46	42.18	–65.96	–15.75	16.95	15.86	–75.65	–15.57	20.48	20.03
10	–79.09	142.77	23.11	15.35	–111.31	79.41	17.73	23.02	–73.21	150.36	18.93	13.29
11	–64.71	152.91	10.95	14.00	–58.86	–33.50	11.19	13.53	–60.57	145.71	10.99	16.15
12	–55.00	–36.69	11.50	13.07	–78.72	–34.38	23.59	15.69	–62.18	–20.58	12.08	14.59
13	–66.88	–21.75	10.89	17.49	–73.89	–34.16	19.77	9.31	–60.56	–26.60	10.47	10.30
14	–89.93	–32.82	25.92	14.79	–59.08	–36.53	9.75	11.47	–66.13	–23.49	11.46	13.03
15	–72.11	–21.68	18.29	26.00	–73.77	–21.61	15.01	14.36	–94.08	–3.18	25.53	19.09
16	–63.92	143.74	19.02	57.94	–76.87	39.53	11.34	13.47	–72.63	143.99	19.51	22.60
17	–61.60	–26.65	24.55	35.71	–51.86	–34.44	13.04	11.74	–54.91	–28.36	24.59	19.96
18	–65.55	–26.16	16.32	15.41	–56.50	–25.98	10.90	12.84	–64.58	–23.26	15.28	14.01
19	–113.61	–24.26	21.61	50.91	–114.06	–37.36	16.21	14.29	–121.10	–23.44	17.82	22.92
20	–120.19	136.48	34.05	50.47	–86.73	–29.24	17.74	14.68	–93.50	144.10	37.04	19.12
21	–68.02	137.27	33.02	52.67	–96.20	12.28	24.34	40.74	60.44	10.14	9.69	26.02

Peptide 3	Cluster 1				Cluster 2				Cluster 3			
Residue	Phi	Psi	SD (Phi)	SD (Psi)	Phi	Psi	SD (Phi)	SD (Psi)	Phi	Psi	SD (Phi)	SD (Psi)
7	–66.40	120.52	10.89	21.55	–66.22	119.92	10.95	20.15	–65.58	140.89	10.57	13.56
8	–137.03	–36.83	22.88	17.25	–138.61	–36.09	21.49	17.15	–90.77	168.68	17.96	13.15
9	–93.45	132.93	24.05	15.39	–90.78	131.86	23.14	14.88	–76.70	–28.60	13.28	18.81
10	–72.18	131.86	22.38	15.95	–73.07	134.50	22.79	17.04	–68.87	151.94	22.31	9.28
11	–68.18	156.44	11.43	45.48	–69.22	157.14	11.72	52.29	–70.03	164.47	10.50	12.43
12	–70.09	–25.15	38.81	25.18	–72.05	–22.90	45.27	59.24	–64.68	–23.82	11.36	18.76
13	–85.26	144.63	34.73	16.80	–83.60	144.90	34.19	17.76	–65.47	141.35	19.50	14.44
14	–66.96	144.61	14.02	11.68	–65.31	134.52	14.51	13.32	–79.11	153.12	18.32	12.20
15	58.43	6.43	9.28	23.75	57.84	9.32	9.51	23.29	61.87	–15.91	8.79	23.19
16	–80.64	152.14	25.04	14.86	–80.41	152.47	25.41	16.50	–71.62	158.04	22.45	14.37
17	–69.91	141.84	19.69	17.06	–69.57	142.73	17.34	15.98	–57.64	129.65	12.16	13.44
18	–63.85	143.15	16.27	15.21	–63.21	144.17	15.30	14.41	–71.42	149.45	13.71	12.29
19	–100.66	161.35	26.97	13.22	–97.02	160.79	27.56	13.43	–71.28	158.39	15.26	13.62
20	–75.66	143.86	20.66	16.51	–76.03	143.95	21.84	16.48	–123.50	143.48	24.94	13.00
21	–71.88	138.85	18.61	26.97	–69.76	139.54	19.15	26.42	–78.27	112.54	16.77	38.84

Supplementary Table 2: Ramachandran plots of SMD simulations of Peptide 2 with different lambda values. The figures were obtained after population reweighting. Energies are reported in kcal/mol. See Supplementary_Table_2

Supplementary Table 3: Ramachandran plots of SMD simulations of Peptide 3 with different lambda values. The figures were obtained after population reweighting. Energies are reported in kcal/mol. See Supplementary_Table_3
