# Supplementary material for: Atomistic molecular dynamics simulations of bioactive engrailed 1 interference peptides (EN1-iPeps)
Source: Oncotarget. 2018 Apr 27;9(32):22383–97. doi: 10.18632/oncotarget.25025 (PMC5976472; doi:10.18632/oncotarget.25025)
Supplement: Supplementary file 2 [file oncotarget-09-22383-s002.pdf]

**Supplementary Table 2: Ramachandran plots of sMD simulations of Peptide 2 with different lambda values**

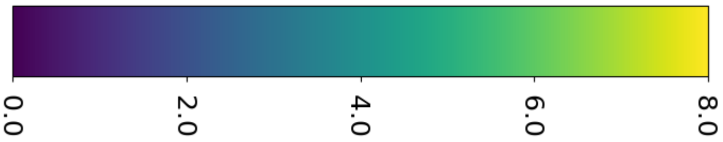


| Residue | ʎ = 0.7 | ʎ = 0.5 |
|---|---|---|
| PRO 7 | 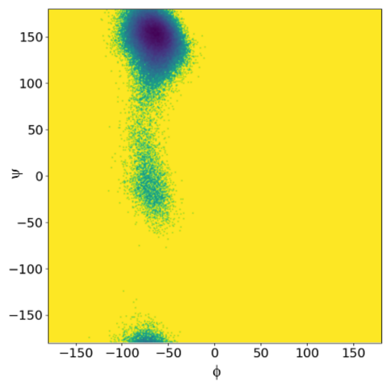  | 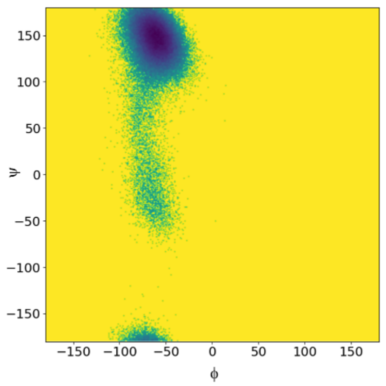  |

| | | |
|---|---|---|
| LEU 8 | 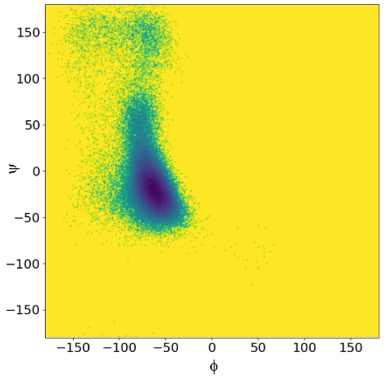 | 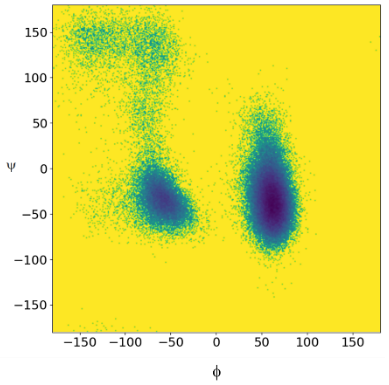 |
| VAL 9 | 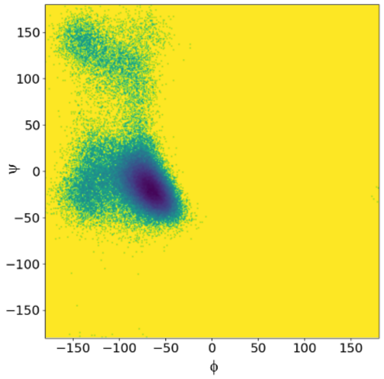 | 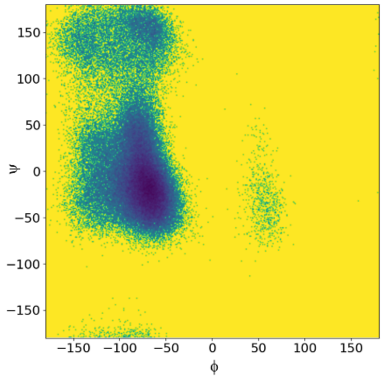 |
| TRP 10 | 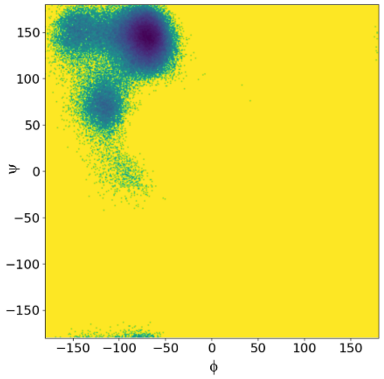 | 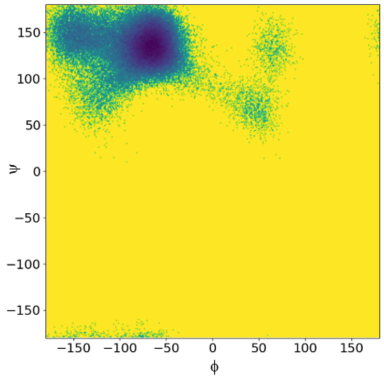 |

PRO 11

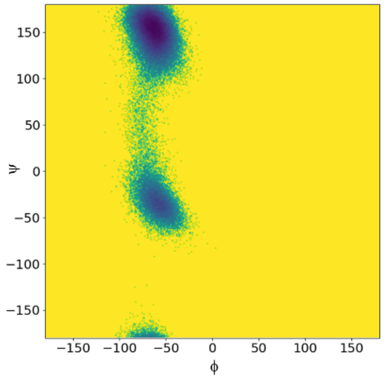


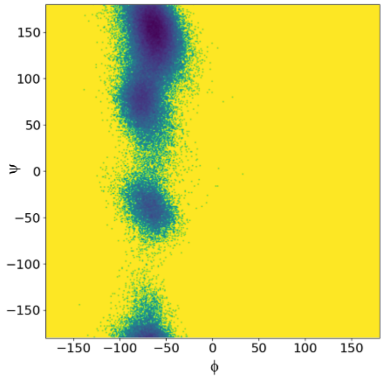


ALA 12

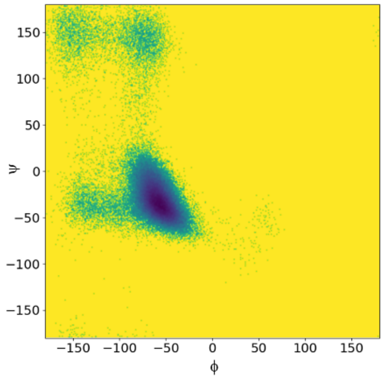


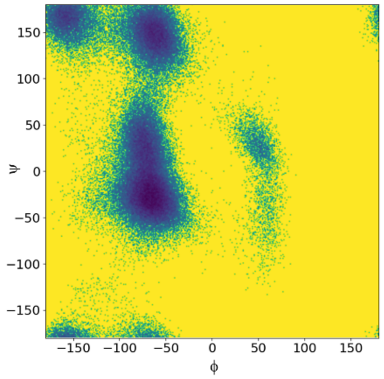


TRP 13

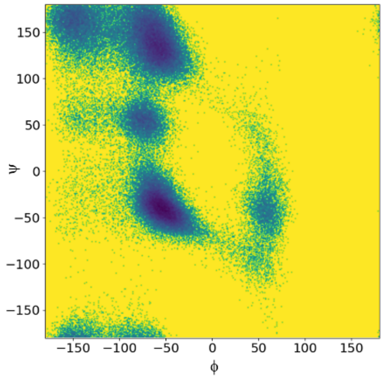


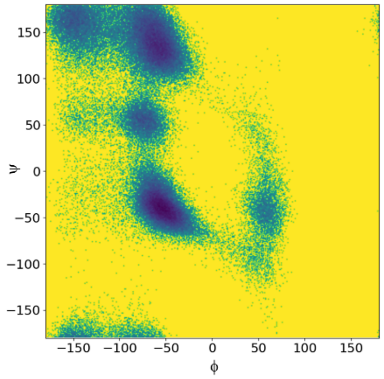

| | | |
|---|---|---|
| VAL 14 | 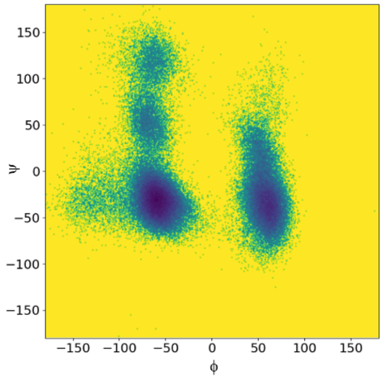 | 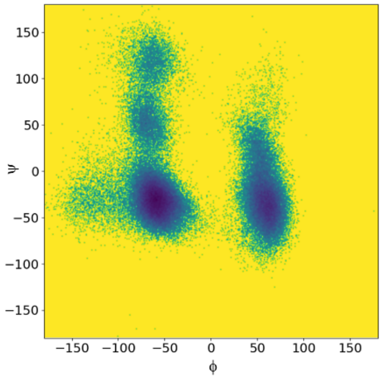 |
| TYR 15 | 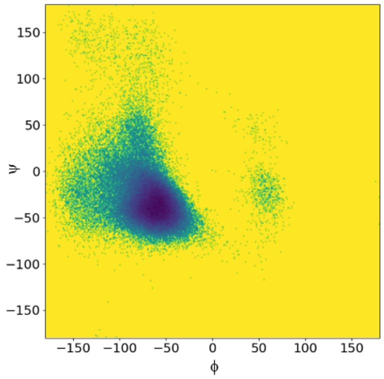 | 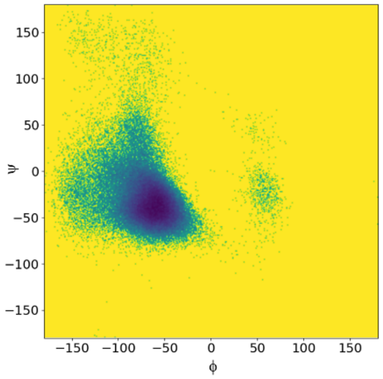 |
| CYS 16 | 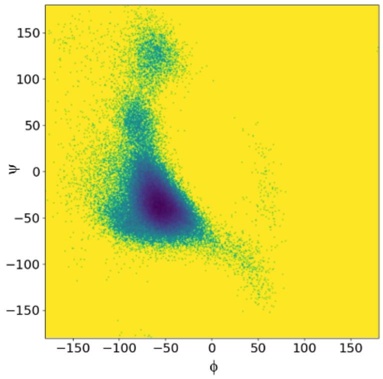 | 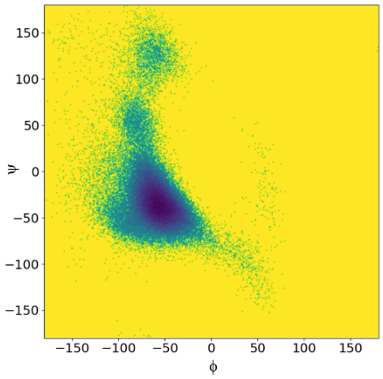 |

THR 17

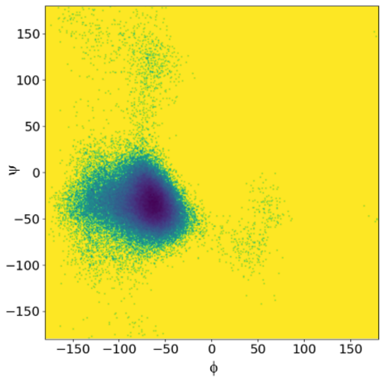


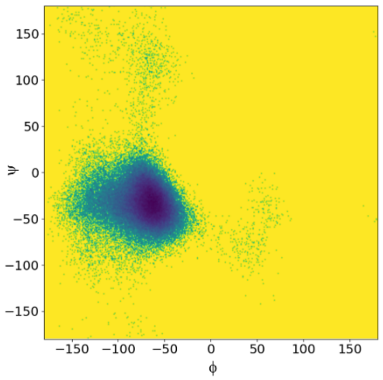


ARG 18

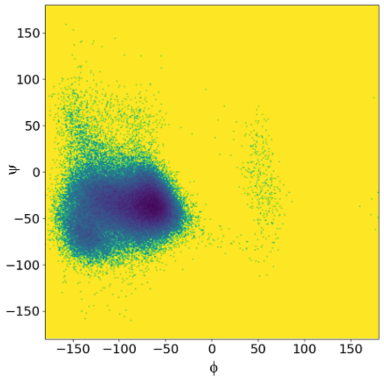


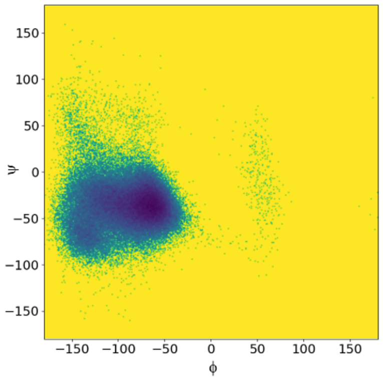


TYR 19

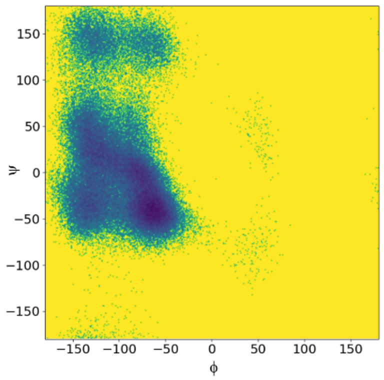


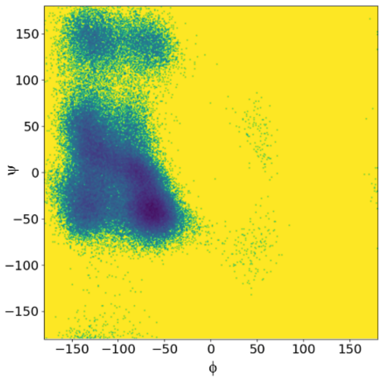

| | | |
|---|---|---|
| SER 20 | 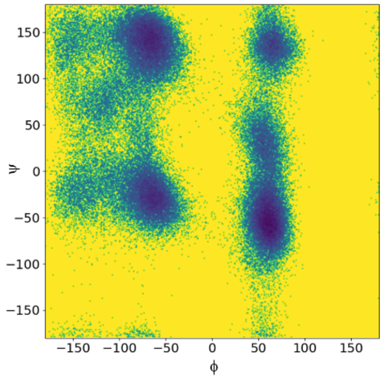 | 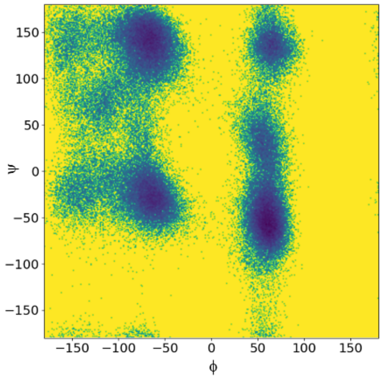 |
| ASP 21 | 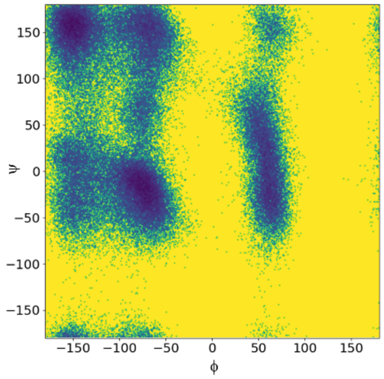 | 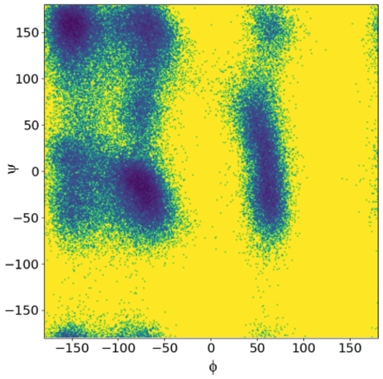 |

The figures were obtained after population reweighting. Energies are reported in kcal/mol.
