# Supplementary material for: Atomistic molecular dynamics simulations of bioactive engrailed 1 interference peptides (EN1-iPeps)
Source: Oncotarget. 2018 Apr 27;9(32):22383–97. doi: 10.18632/oncotarget.25025 (PMC5976472; doi:10.18632/oncotarget.25025)
Supplement: Supplementary file 3 [file oncotarget-09-22383-s003.pdf]

Supplementary Table 3: Ramachandran plots of sMD simulations of Peptide 3 with different lambda values

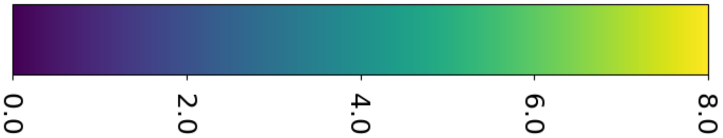

Residue	ʎ = 0.7	ʎ = 0.5
PRO 7	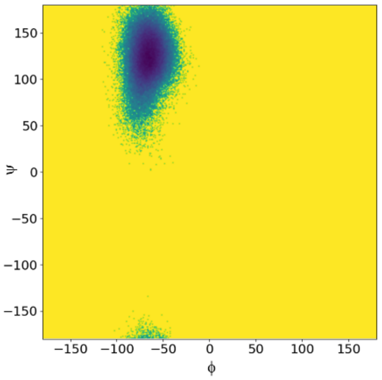	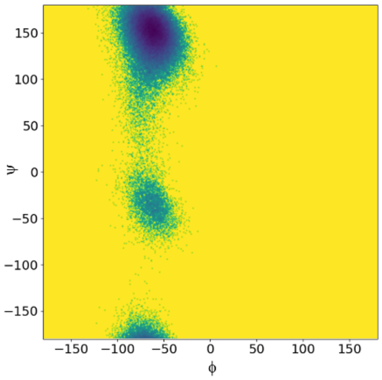

<table>
<tr><td>LEU 8</td><td>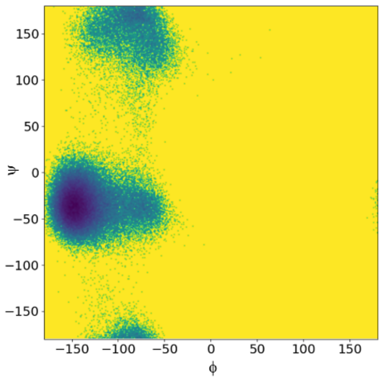</td><td>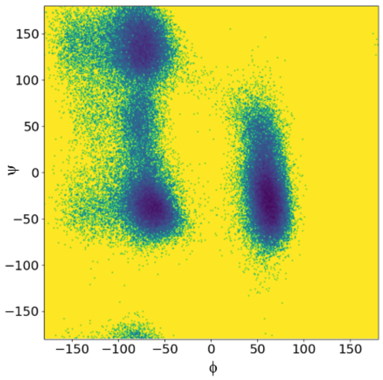</td></tr>
<tr><td>VAL 9</td><td>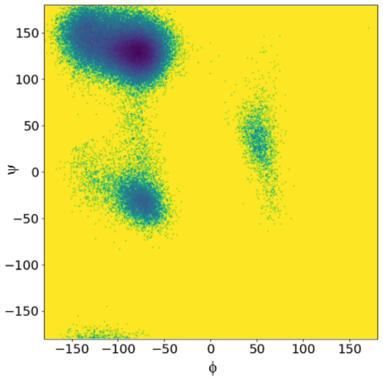</td><td>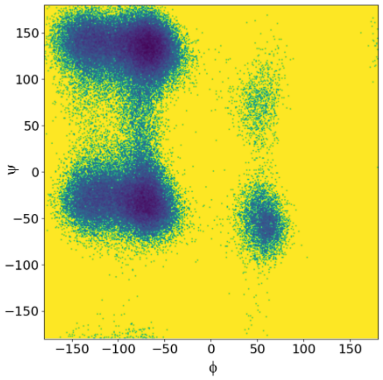</td></tr>
<tr><td>ALA 10</td><td>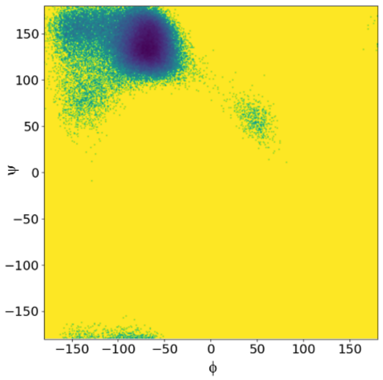</td><td>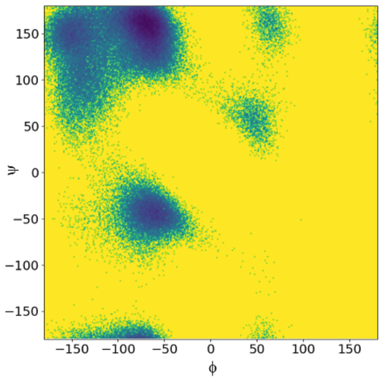</td></tr>
</table>

PRO 11

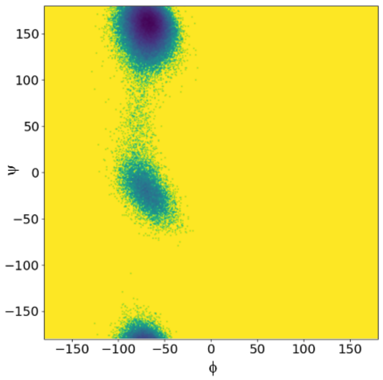

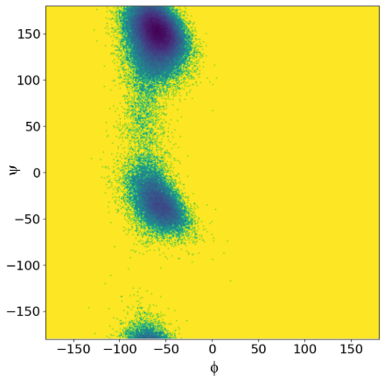

ALA 12

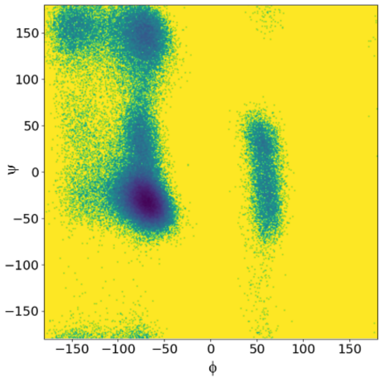

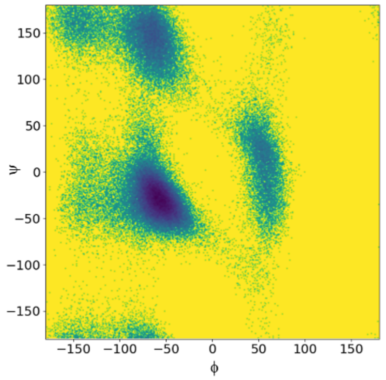

ALA 13

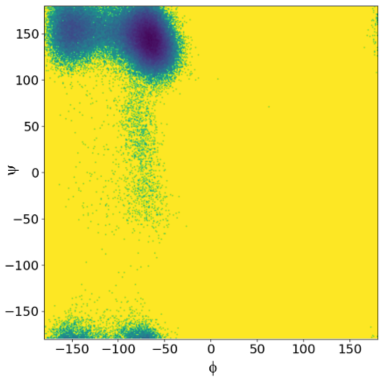

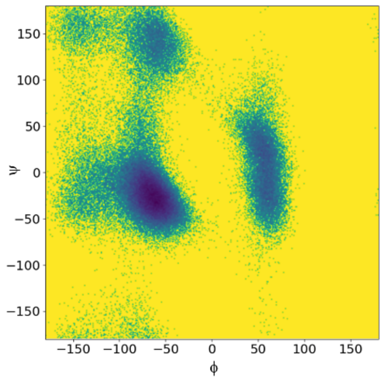

VAL 14	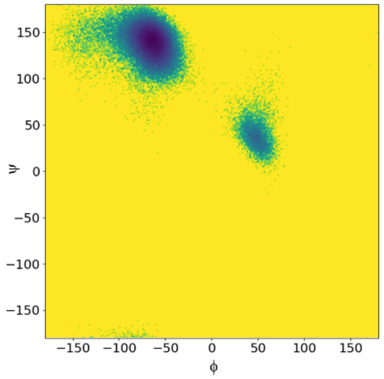	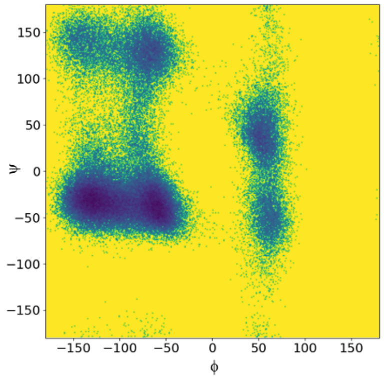
TYR 15	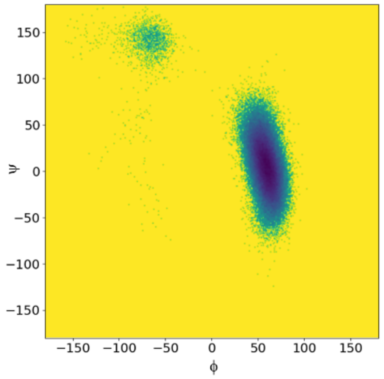	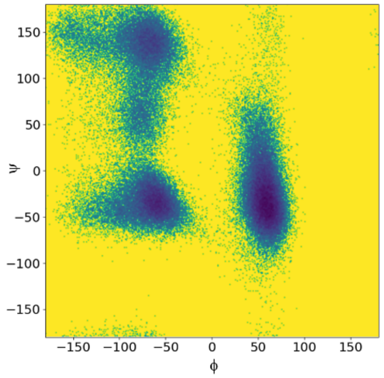
CYS 16	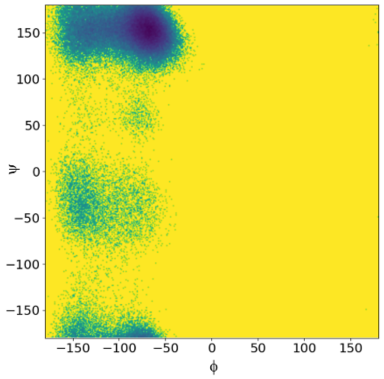	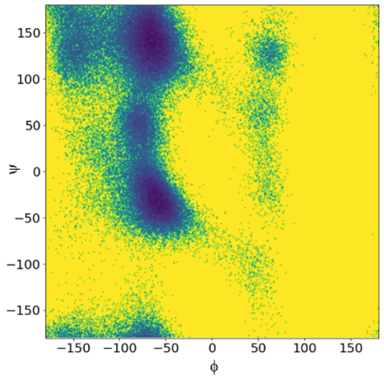

THR 17	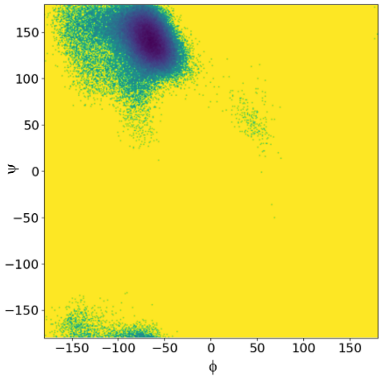	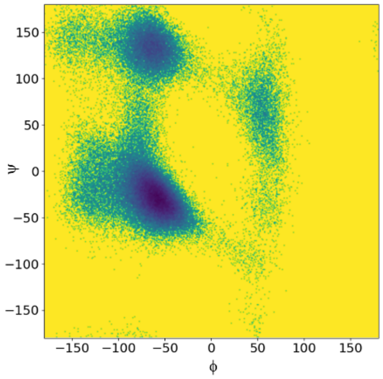
ARG 18	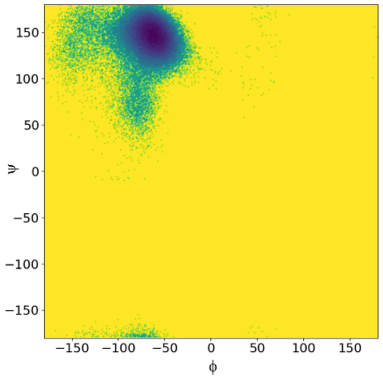	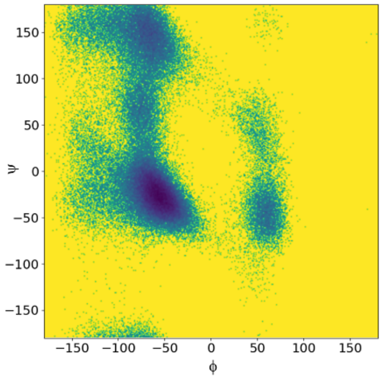
TYR 19	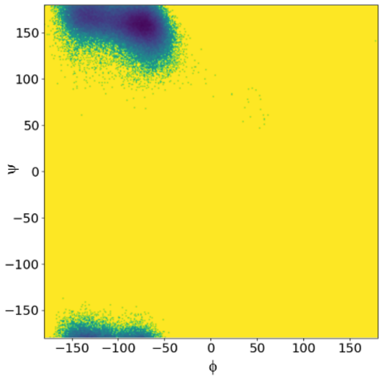	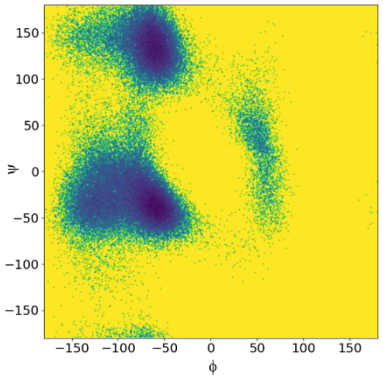

SER 20	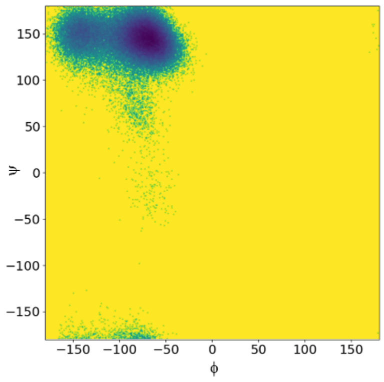	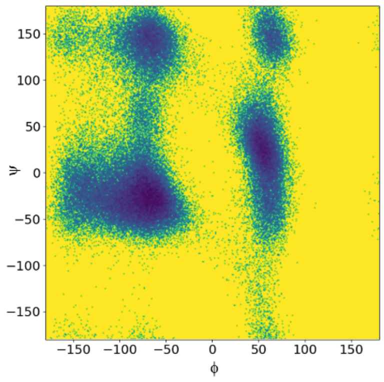
ASP 21	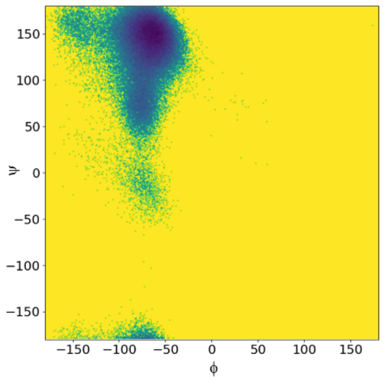	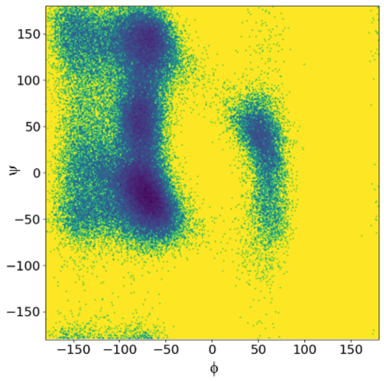

The figures were obtained after population reweighting. Energies are reported in kcal/mol.
